# CRISPR/Cas9 screens identify LIG1 as a sensitizer of PARP inhibitors in castration-resistant prostate cancer

**DOI:** 10.1172/JCI179393

**Published:** 2024-12-24

**Authors:** Giulia Fracassi, Francesca Lorenzin, Francesco Orlando, Ubaldo Gioia, Giacomo D’Amato, Arnau S. Casaramona, Thomas Cantore, Davide Prandi, Frédéric R. Santer, Helmut Klocker, Fabrizio d’Adda di Fagagna, Joaquin Mateo, Francesca Demichelis

**Affiliations:** 1 Department of Cellular, Computational and Integrative Biology (CIBIO), University of Trento, Trento, Italy.; 2 Institute of Molecular Genetics, National Research Council, Pavia, Italy.; 3 IFOM ETS–The AIRC Institute of Molecular Oncology, Milan, Italy.; 4 Vall d’Hebron Institute of Oncology (VHIO), Vall d’Hebron University Hospital Campus, Barcelona, Spain.; 5 Department of Urology, Division of Experimental Urology, Medical University of Innsbruck, Innsbruck, Austria.

**Keywords:** Oncology, DNA repair, Prostate cancer

## Abstract

PARP inhibitors (PARPi) have received regulatory approval for the treatment of several tumors, including prostate cancer (PCa), and demonstrate remarkable results in the treatment of castration-resistant prostate cancer (CRPC) patients characterized by defects in homologous recombination repair (HRR) genes. Preclinical studies showed that DNA repair genes (DRG) other than HRR genes may have therapeutic value in the context of PARPi. To this end, we performed multiple CRISPR/Cas9 screens in PCa cell lines using a custom sgRNA library targeting DRG combined with PARPi treatment. We identified DNA ligase 1 (*LIG1*), essential meiotic structure-specific endonuclease 1 (*EME1*), and Fanconi anemia core complex associated protein 24 (*FAAP24*) losses as PARPi sensitizers and assessed their frequencies from 3% to 6% among CRPC patients. We showed that concomitant inactivation of LIG1 and PARP induced replication stress and DNA double-strand breaks, ultimately leading to apoptosis. This synthetic lethality (SL) is conserved across multiple tumor types (e.g., lung, breast, and colorectal), and its applicability might be extended to LIG1-functional tumors through a pharmacological combinatorial approach. Importantly, the sensitivity of *LIG1*-deficient cells to PARPi was confirmed in vivo. Altogether, our results argue for the relevance of determining the status of *LIG1* and potentially other non-HRR DRG for CRPC patient stratification and provide evidence to expand their therapeutic options.

## Abstract 2

LIG1 inactivation confers sensitivity to PARP inhibitor treatment in advanced prostate cancer

## Introduction

Prostate cancer (PCa) is a clinically and genetically heterogeneous disease entity that exhibits a wide spectrum of clinical behaviors, from relatively indolent to metastatic progression and lethality. Castration-resistant prostate cancer (CRPC) is an advanced and lethal disease that arises after the development of resistance to conventional androgen receptor signaling inhibitors (ARSI), and for which therapeutic options are still limited.

Synthetic lethality–based (SL-based) approaches represent a valuable strategy to identify novel therapeutic opportunities for cancer treatment. The initial discovery of the SL interaction between *BRCA1/2* and *PARP1* in breast cancer (BRCA) and ovarian cancer (OV) is the pivotal example of the bench to bedside translational potential of SL and has paved the way for the use of PARP inhibitors (PARPi) in other tumor types characterized by homologous recombination repair (HRR) gene mutations (1, 2).

In the last decade, several studies delineated the genomic landscape of both primary and advanced PCa, contributing to defining molecular subclasses and expanding the therapeutic options for PCa treatment (3–6). Androgen receptor (*AR*) amplifications and locus rearrangements are the most frequent aberrations found in CRPC and are associated with resistance to therapy. Among others, high frequency aberrations in CRPC include homozygous deletion or loss-of-function mutations in *PTEN* and alterations in *TP53* and *RB1* genes (4–7). Somatic and/or germline aberrations in DNA repair genes (DRG) involved in the HRR pathway – including *BRCA1*/*2*, *ATM*, *PALB2*, *CHEK2*, and *FANCA* – have also been detected in the genome of 20%–27% of CRPC patients (4, 6, 8, 9). Furthermore, the incidence of both somatic and germline DRG defects increases from localized PCa (10%) to CRPC (3, 4, 6, 8, 9), and inherited DRG variants (especially in *BRCA2*) associate with high risk of developing more aggressive PCa (10–13). More recently, a comprehensive genomic characterization of PCa with the combined and uniform analysis of 1,013 primary and advanced PCa samples identified a long-tail distribution of genes mutated at a frequency below 3%, which included DRG beyond the classical HRR genes (6). Although these genes have a low frequency of aberration, they might still be relevant for a significant fraction of patients if considering the high incidence of PCa and warrant further investigation.

The observation that CRPC patients harbor HRR gene mutations prompted the use of PARPi in this context. Multiple clinical trials have demonstrated the efficacy and led to FDA and EMA approval of olaparib (OLA) and rucaparib for the treatment of CRPC patients with mutations in a subset of HRR genes (14–18). Still, questions have been raised about the patients’ enrollment criteria given that the biomarkers defined for the 2 PARPi are different (14 HRR genes for OLA, *BRCA1*/*2* for rucaparib) and PARPi sensitivity is heterogeneous among patients with HRR gene alterations (14–17, 19). Moreover, the observations that a subset of biomarker-negative patients benefits from PARPi treatment and that the combination of PARPi and ARSI — abiraterone or enzalutamide (ENZA) — without biomarker-based selection improves the prognosis of CRPC patients suggest that other DRG might confer vulnerability to PARPi (14, 20–22). Similarly, preclinical studies indicate that defects in genes (e.g., *RNASEH2B*, *CHD1L*, and *FEN1*) involved in DNA repair pathways other than HRR could have therapeutic potential when combined with PARPi treatment (23–26).

To identify novel DRG aberrations associated with PARPi sensitivity in CRPC, we performed multiple CRISPR/Cas9 genotoxic screens in BRCA1/2 proficient PCa cell lines treated with the 2 PARPi OLA and talazoparib (TALA) and using a custom sgRNA library targeting 356 DRG belonging to 7 different DNA repair pathways. This enabled us to nominate DNA ligase I (*LIG1*), essential meiotic structure-specific endonuclease 1 (*EME1*), and Fanconi anemia core complex associated protein 24 (*FAAP24*) as vulnerabilities associated with PARPi sensitivity. We validated the SL interaction between *LIG1* and *PARP* in multiple cancer models and in PCa xenografts and provided initial evidence supporting the efficacy of combined LIG1 and PARP pharmacological inhibition. Altogether, we identified *LIG1* and other non-HRR genes as potential biomarkers that might help to better stratify CRPC patients.

## Results

### Custom CRISPR/Cas9 screens identified LIG1, EME1, and FAAP24 losses as associated with PARPi sensitivity in PCa cells.

**Figure 1:**
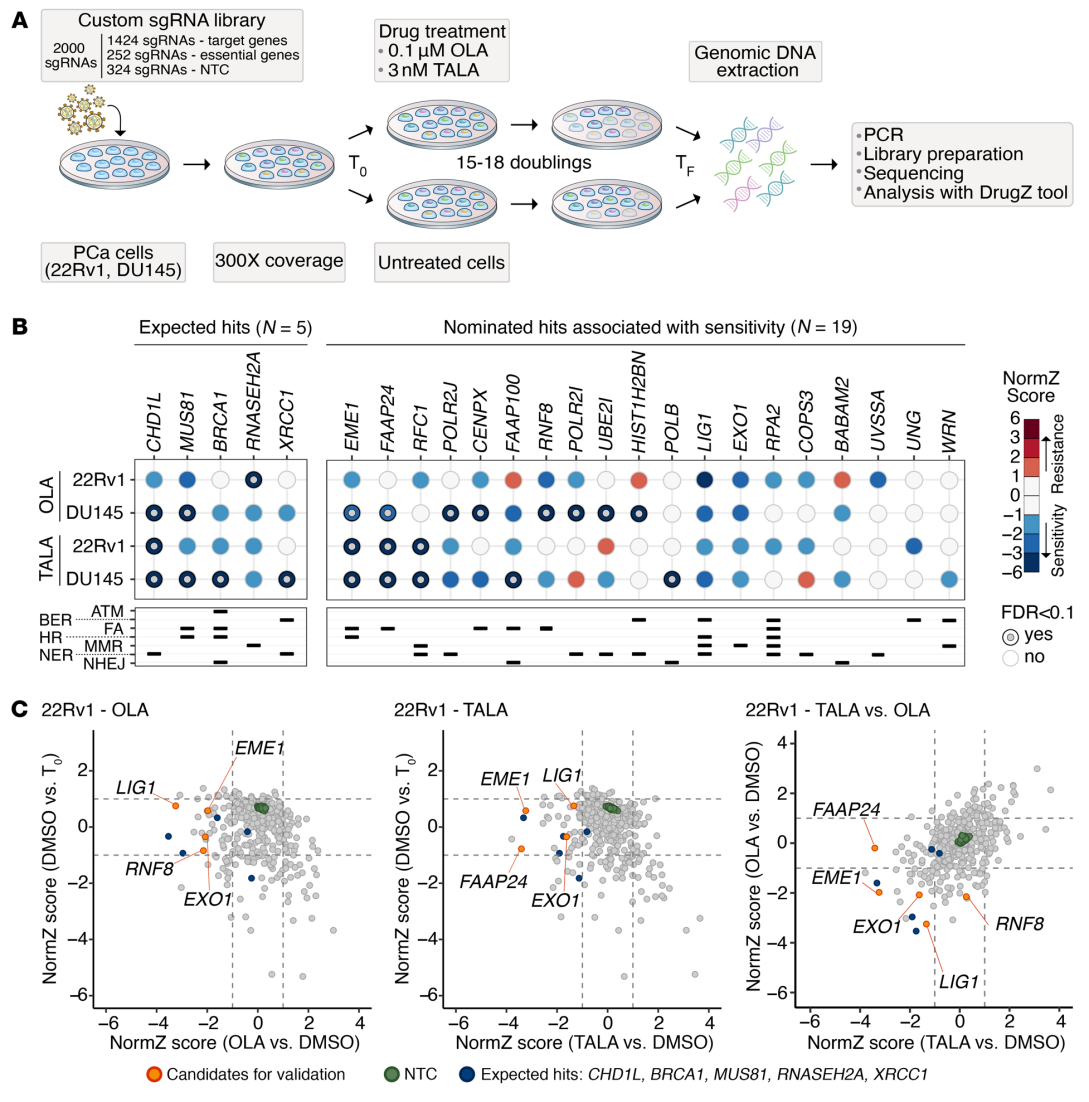
CRISPR/Cas9 screens combined with PARPi highlighted DRG-related vulnerabilities in PCa cells. (**A**) Schematic of the CRISPR/Cas9 genotoxic dropout screens (DrugZ tool from ref. 30). (**B**) Bubble plot of the CRISPR/Cas9 screen results. Expected hits include genes previously associated with PARPi sensitivity (1, 25, 26, 31–34). Nominated hits were selected based on FDR < 0.1 (gray circle and border) or NormZ score lower than –1 in at least one condition. DRG function in the various DNA repair pathways is reported. (**C**) Scatter plots of the CRISPR/Cas9 screen results in 22Rv1 treated with OLA (0.1 μM) or TALA (3 nM). Dashed lines indicate the +1 and –1 NormZ scores. Expected hits (blue) as in **B**. Genes that demonstrated sensitivity to PARPi in both cell lines were selected for in vitro validation (orange). NTC, nontargeting control; T_0_, start of treatment; T_F_, end of treatment; ATM, ataxia-telangiectasia mutated; BER, base excision repair; FA, Fanconi anemia; HR, homologous recombination; MMR, mismatch repair; NER, nucleotide excision repair; NHEJ, non-homologous end joining.

To identify gene losses associated with increased sensitivity to PARPi treatment, we performed CRISPR/Cas9 knockout (KO) screens combined with the administration of 2 PARPi (OLA or TALA). We used 22Rv1 and DU145 PCa cell lines and a custom sgRNA pooled library (Supplemental Table 1; supplemental material available online with this article; https://doi.org/10.1172/JCI179393DS1) targeting 356 DRG and including sgRNAs against 63 essential genes (27–29) and 324 nontargeting control (NTC) sgRNAs as positive and negative controls, respectively (Figure 1A). 22Rv1 and DU145 PCa cell lines showed similar responses to PARPi and were selected based on the AR status (22Rv1 cells are AR positive while DU145 cells are AR negative) to mimic CRPC states and the absence of biallelic loss-of-function alterations in DRG previously associated with PARPi sensitivity (Supplemental Figure 1, A and B).

For the screens, 22Rv1 and DU145 single clones with a confirmed Cas9 activity higher than 70% (Supplemental Figure 1C) were isolated and transduced with the lentiviral custom sgRNA library (multiplicity of infection [MOI] ~ 0.3) (Figure 1A). After selection, a fraction of cells was collected to represent the initial population (T_0_) while the remaining cells were divided into control (DMSO) and treated (OLA and TALA) groups. All 3 groups were grown for 15–18 population doublings, and after DNA extraction, sgRNA cassette amplification, and sequencing (average coverage of 400–500×, Supplemental Figure 1C), data were analyzed using the DrugZ software (30) to calculate normalized *z* (NormZ) scores (Supplemental Tables 2–7).

As expected, essential genes showed a NormZ score significantly lower than NTC and target genes, indicating that their KO negatively affected the fitness of 22Rv1 and DU145 cells, while NTC had no effect on cellular fitness with a NormZ score around 0 (Supplemental Figure 1D).

**Figure 2:**
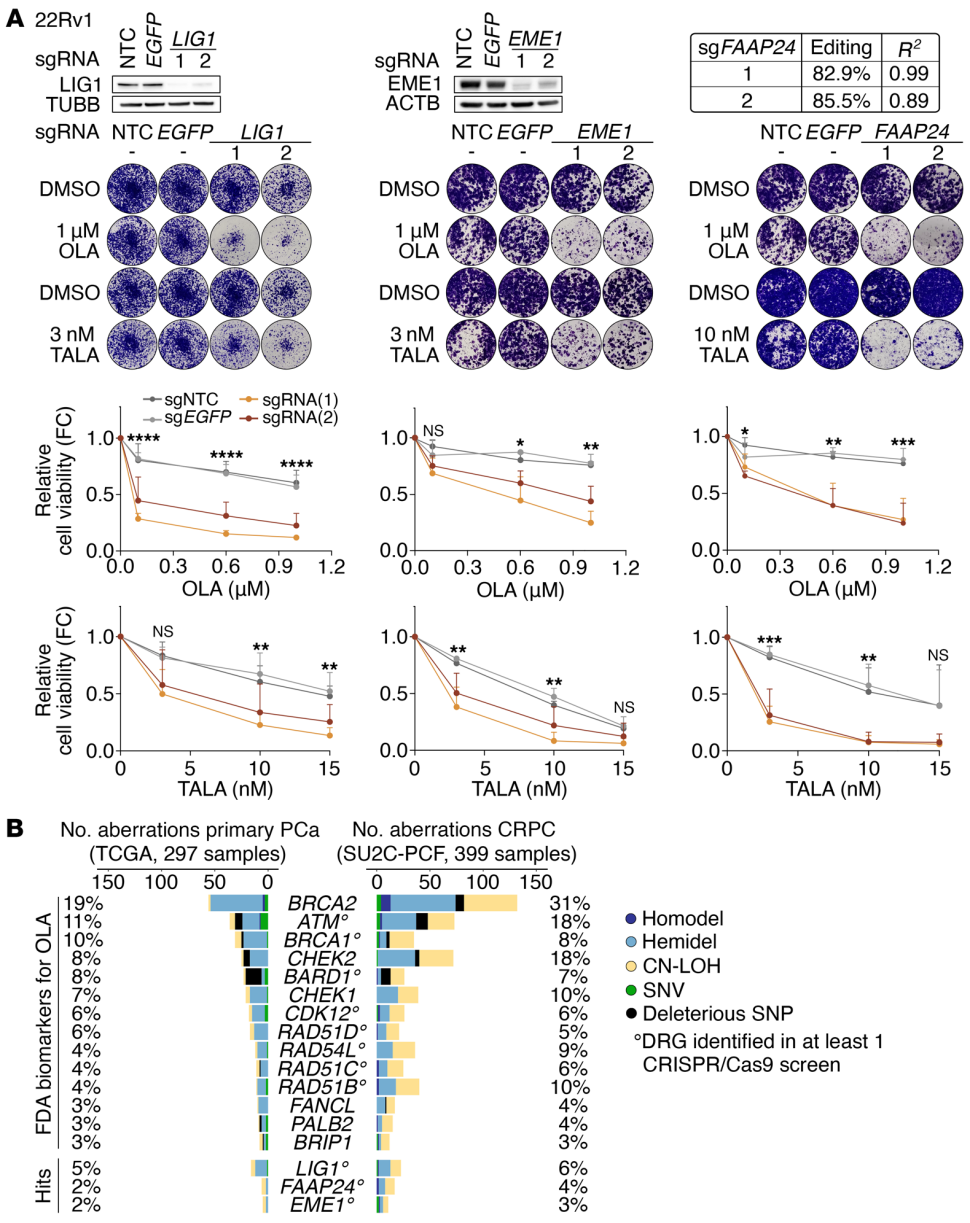
*LIG1*, *EME1*, and *FAAP24* KO sensitize cells to PARPi treatment. (**A**) Immunoblots or TIDE analysis of the indicated DRG KO and representative images of the crystal violet assays with the corresponding quantifications. 22Rv1 cells transduced with the indicated sgRNAs were treated with OLA or TALA for 12–15 days (DMSO was used as control). Data are presented as mean + SD (*n* = 3 biological replicates). *P* values were determined using the 2-way ANOVA and Bonferroni’s multiple comparisons test on control (sgNTC and sg*EGFP*) and sg*LIG1* samples. *P ≤ 0.05; **P ≤ 0.01; ***P ≤ 0.001; ****P ≤ 0.0001. Two replicates of the *EME1* and *FAAP24* OLA- and TALA-related experiments were conducted concurrently and share the same controls. (**B**) Incidence of aberrations for the DRG included in the list of FDA-approved biomarkers for OLA and for the validated hits in the TCGA (44) and SU2C-PCF (5) cohorts. SNV, single nucleotide variant.

Next, NormZ values were calculated for PARPi-treated samples compared with DMSO treatment (OLA or TALA versus DMSO) and were combined with the NormZ score (DMSO versus T_0_) to identify nonessential genes linked to treatment sensitization. We nominated 24 genes associated with sensitivity to OLA or TALA treatment, 5 of which (i.e., *CHD1L*, *BRCA1*, *MUS81*, *RNASEH2A*, and *XRCC1*) have been previously reported as associated with increased response to PARPi (Figure 1, B and C, and Supplemental Figure 1E) (1, 25, 26, 31–34). From the remaining 19 DRG, we selected *EME1*, *FAAP24*, *RNF8*, *LIG1*, and *EXO1* for in vitro validation. *EME1*, *LIG1*, and *EXO1* were associated with both OLA and TALA sensitivity, *RNF8* was a common candidate for OLA treatment, and *FAAP24* was identified as a hit for TALA (Figure 1C). Survival assays using 22Rv1 control and KO cells treated with PARPi showed negative or inconsistent results for *RNF8* and *EXO1*, respectively (Supplemental Figure 2, A and B), whereas they confirmed the markedly increased sensitivity of *LIG1*-, *FAAP24*-, and *EME1*-KO cells to OLA and TALA (corrected *P* values < 0.05) (Figure 2A).

*LIG1* encodes for the DNA ligase I and is implicated in DNA replication, recombination, and repair where it seals Okazaki fragments and ligates nicks generated during DNA repair (35, 36). EME1 is the regulatory subunit of an endonuclease complex (MUS81-EME1) involved in the resolution of DNA intermediates during recombination and replication (37–40). FAAP24 is associated with the recruitment of the Fanconi anemia complex and the regulation of ATR-CHK1 checkpoint signaling (41, 42).

To explore the clinical relevance of the validated candidates, we examined the incidence of germline and somatic loss-of-function alterations in cohorts of primary PCa (The Cancer Genome Atlas [TCGA]) and CRPC (Stand Up to Cancer [SU2C]-PCF) samples (5, 43), upon in-house processing (44). As a comparison, we included the aberration frequency of the DRG included in the list of FDA-approved biomarkers for OLA. *LIG1*, *EME1*, and *FAAP24* are characterized by a low incidence of aberration that is, however, comparable with some FDA-approved DRG (Figure 2B). *LIG1* emerged as the most frequently aberrant gene among the candidates in both TCGA (5%) and SU2C-PCF (6%) cohorts. Additionally, we found support for the SL interaction between *LIG1* and *PARP1* and *EME1* and *PARP1* by analyzing the coexpression of *LIG1*, *EME1*, or *FAAP24* and *PARP1* in relation to the tumor, node, metastasis (TNM) stage of PCa samples (*P* value for *LIG1*-*PARP1* < 0.001; *P* value for *EME1*-*PARP1* < 0.05) (Supplemental Figure 2C).

Overall, the results of the CRISPR/Cas9 screens, the in vitro validation, and the analysis of patient-derived genomic and transcriptomic data nominate 2 SL interactions (*LIG1*-*PARP* and *EME1*-*PARP*) with translational potential and support the selection of *LIG1* for further investigations.

### Combined LIG1 loss and PARP inhibition induce DNA damage and apoptosis in PCa cells.

**Figure 3:**
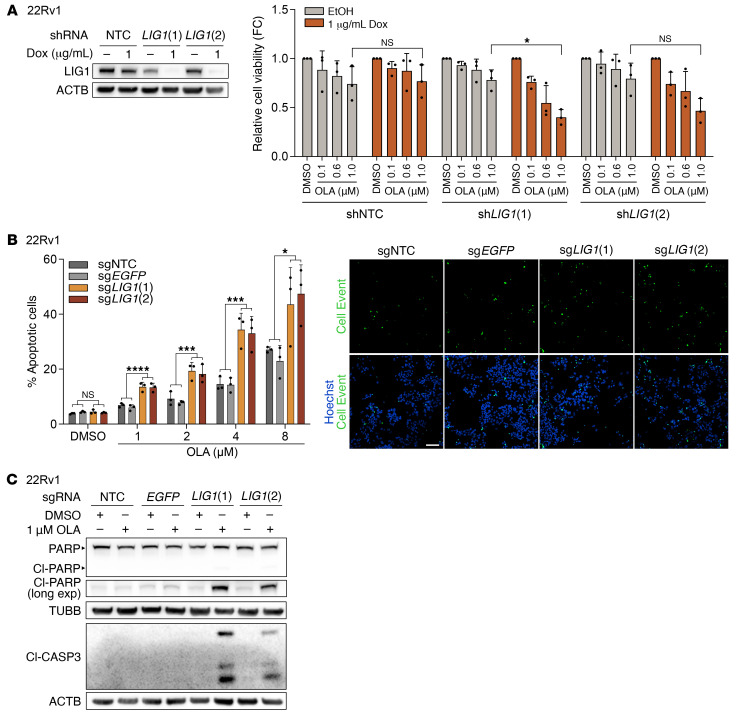
LIG1 loss combined with OLA treatment induces apoptosis in 22Rv1 cells. (**A**) Immunoblot of LIG1 protein levels and cell viability measured with CCK8 assay in 22Rv1 cells transduced with the indicated inducible shRNAs. Cells were treated for 12 days with ethanol (EtOH), as control, or Dox to induce shRNA expression, and with DMSO, as control, or OLA. Data are presented as mean ± SD (*n* = 3 biological replicates). *P* values were determined using a 2-way ANOVA and Bonferroni’s multiple-comparisons test. (**B**) Percentage of apoptotic cells measured by CellEvent caspase-3/7 assay in 22Rv1 cells transduced with the indicated sgRNA and treated with DMSO, as control, or OLA for 5 days. Data are presented as mean ± SD (*n* = 3 biological replicates). *P* values were determined using 2-way ANOVA and Bonferroni’s multiple-comparisons test on control (sgNTC and sg*EGFP*) and sg*LIG1* samples. Images are representative of 22Rv1 cells treated with 4 μM OLA. Scale bar: 100 μm. (**C**) Immunoblot of PARP, cleaved PARP (Cl-PARP), cleaved caspase-3 (Cl-CASP3), and ACTB (used as loading control) in 22Rv1 transduced with the indicated sgRNAs and treated for 3 days with DMSO, as control, or OLA. *P ≤ 0.05; **P ≤ 0.01; ***P ≤ 0.001; ****P ≤ 0.0001. Long exp: longer exposure.

To further validate the SL interaction between *LIG1* and *PARP*, we monitored cell proliferation and detected no strong differences between untreated *LIG1*-KO or knockdown (KD) 22Rv1 cells and controls (Supplemental Figure 3, A and B), confirming that LIG1 is not essential and that its loss does not confer proliferative advantages in vitro. In line with our previous observations, shRNA-mediated depletion of *LIG1* combined with OLA treatment significantly decreased cell survival (Figure 3A) (corrected *P* value for sg*LIG1* [1] treated with doxycycline (Dox) and 1 μM OLA < 0.05, corrected *P* value for sg*LIG1* [2] treated with Dox and 1 μM OLA = NS). LIG1 and PARP SL was also confirmed in DU145 (Supplemental Figure 3C). Next, we tested whether *LIG1* loss and PARPi treatment led to cell death by performing CellEvent caspase-3/7 assay and immunoblot analyses. We observed a significant increase in the percentage of cleaved caspase-3/7–positive cells (corrected *P* values for sg*LIG1* treated with OLA < 0.05) and a strong activation of caspase-3 and PARP through the detection of their cleaved forms in *LIG1*-KO cells upon PARPi administration (Figure 3, B and C), while the effects on control cells treated with OLA were mild. These findings were further confirmed by FACS analysis of annexin-V and propidium iodide (PI) (Supplemental Figure 3D). Similar results were obtained using TALA (Supplemental Figure 3, E–G). Altogether, these results indicate that apoptosis is induced in *LIG1*-KO cells treated with PARPi.

**Figure 4:**
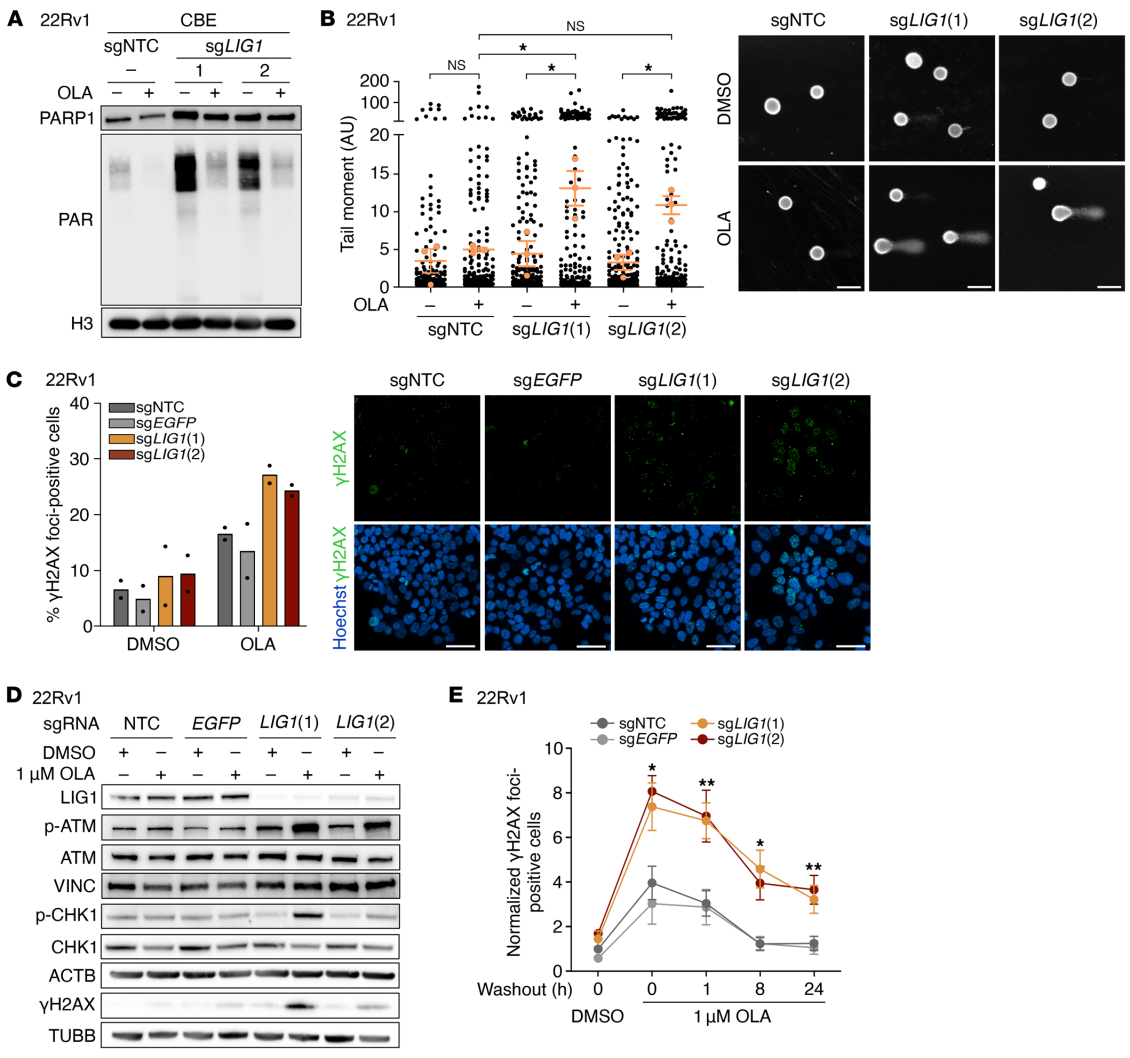
LIG1 loss combined with OLA treatment induces DNA damage in 22Rv1 cells. (**A**) Immunoblot of PARP1, PAR, and H3 (used as loading control) in the chromatin-bound extract (CBE) of 22Rv1 transduced with the indicated sgRNAs and treated for 3 days with DMSO, as control, or OLA. (**B**) Comet tail moment measured by alkaline comet assay in 22Rv1 transduced with the indicated sgRNAs and treated as in **A**. The orange dots and bars indicate the mean value of each replicate and the mean ± SEM of the 3 experiments, respectively. *P* values were determined using 1-way ANOVA and Tukey’s multiple-comparisons test. Images are representative of the comet assays. Scale bars: 100 μm. (**C**) Quantification of cells with 5 or more γH2AX foci measured by immunofluorescence. Data are presented as mean (*n* = 2 biological replicates). Images are representative of γH2AX (green) and nucleus (Hoechst, blue) immunostaining in 22Rv1 transduced with the indicated sgRNAs and treated as in **A**. Scale bars: 50 μm. (**D**) Immunoblot of LIG1, p-ATM, ATM, VINC (used as loading control), p-CHK1, CHK1, ACTB (used as loading control), γH2AX, and TUBB (used as loading control) in 22Rv1 transduced with the indicated sgRNAs and treated as in **A**. (**E**) Percentage of cells with 10 or more γH2AX foci measured by immunofluorescence after OLA washout. 22Rv1 transduced with the indicated sgRNAs were treated with 1 μM OLA for 3 days and then grown without treatment for 0, 1, 8 or 24 hours. Data are presented as mean ± SEM (*n* = 3 biological replicates). *P* values were determined using 2-way ANOVA and Bonferroni’s multiple-comparisons test on control (sgNTC and sg*EGFP*) and sg*LIG1* samples. *P ≤ 0.05; **P ≤ 0.01.

To investigate the possible mechanism underlying the *LIG1* and *PARP* SL, we first checked the impact of *LIG1* KO on PARP activity. We observed that LIG1-deficient cells recruit more PARP1 on chromatin with and without PARPi treatment compared with control cells and that this is accompanied by increased levels of PARylation (PAR) in untreated conditions (Figure 4A and Supplemental Figure 4A). Given the double role of LIG1 in DNA damage repair and DNA replication (35, 36), we hypothesized that the absence of LIG1 might result in DNA damage, which is promptly repaired thanks to the activity of PARP. However, upon chemical inhibition of PARP, unrepaired DNA breaks accumulate, leading to apoptosis. To test this hypothesis, we investigated DNA damage induction and activation of specific DNA damage response and repair pathways upon LIG1 and PARP inactivation. Via alkaline comet assay, we found that treatment with OLA significantly increased DNA breaks specifically in 22Rv1 *LIG1*-KO cells (corrected *P* values for sg*LIG1* (1) and (2) treated with OLA <0.05) (Figure 4B). Moreover, an increase in the percentage of γH2AX foci–positive cells was detected in *LIG1*-KO cells treated with OLA (Figure 4C). Activation of the DNA damage response as evidenced by augmented phosphorylation of ATM and CHK1 in 22Rv1 *LIG1*-KO cells treated with PARPi was consistent with the induction of DNA double-strand breaks and replication stress by LIG1 and PARP inactivation (Figure 4D and Supplemental Figure 4B). Further supporting the presence of replication defects in cells with nonfunctional LIG1 and PARP, we detected an accumulation of γH2AX in S and G_2_/M phase cells (Supplemental Figure 4C).

To examine the activity of DNA damage repair pathways, we performed 53BP1 immunofluorescence and employed an HRR-EGFP assay to assess the functionality of nonhomologous end joining (NHEJ) and homologous recombination (HR), respectively. We found no differences between 22Rv1 *LIG1*-KO and control cells (Supplemental Figure 4, D–F). Next, we analyzed the ability of LIG1-deficient cells to recover from OLA-induced DNA damage. Following OLA washout, although the percentage of γH2AX foci–positive cells remained higher in *LIG1*-KO samples compared with control cells at the latest time point (corrected *P* values for sg*LIG1* after OLA washout <0.05), it decreased over time following the same kinetics in all samples. This indicates that the absence of LIG1 does not compromise the capability of cells to resolve DNA damage (Figure 4E).

Taken together, our data indicate that PARP activity is indispensable for LIG1-deficient cells to signal and resolve DNA damage. In the absence of functional PARP, these cells accumulate extensive unrepaired DNA lesions and ultimately undergo apoptosis.

### The SL between LIG1 and PARP has therapeutic potential in multiple tumor models.

**Figure 5:**
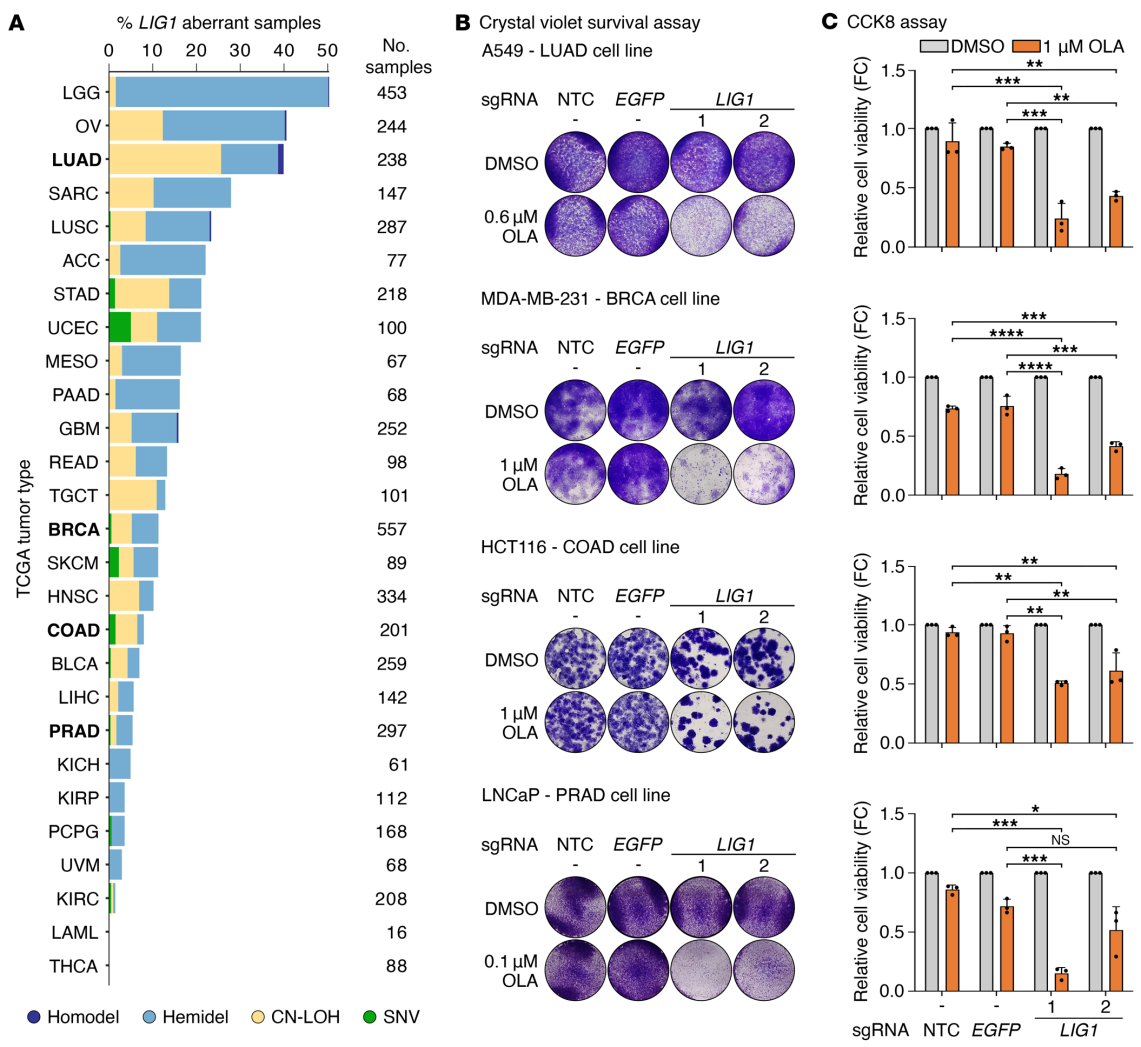
*LIG1* and *PARP* are synthetically lethal in multiple tumor types. (**A**) Incidence of *LIG1* loss-of-function aberrations across 27 tumor types (TCGA, *n* = 4378; ref. 44). (**B**) Representative images of the crystal violet assays in A549, MDA-MB-231, HCT116 and LNCaP cells transduced with the indicated sgRNAs and treated with DMSO, as control, or OLA at different concentrations for 8–12 days. Single images correspond to a well in either a 24-well plate (LNCaP, well diameter: 1.56 cm) or a 48-well plate (A549, MDA-MB-231, and HCT116, well diameter: 1.13 cm). (**C**) Cell viability measured with CCK8 assays in cell lines as in **B**. Data are presented as mean + SD (*n* = 3 biological replicates). *P* values were determined using 1-way ANOVA and Tukey’s multiple-comparisons test. *P ≤ 0.05; **P ≤ 0.01; ***P ≤ 0.001; ****P ≤ 0.0001.

We questioned whether the SL between *LIG1* and *PARP* is translatable to various cancer types, also in keeping with recent CRISPR/Cas9 genome-wide screens in BRCA and OV cell lines treated with PARPi that reported *LIG1* among the genes giving sensitivity to the treatment, although this association was not pursued further (45, 46). We analyzed pan-cancer TCGA genomic and transcriptomic data (44) and detected *LIG1* loss-of-function alterations with concomitant decrease in expression in multiple tumor types (Figure 5A and Supplemental Figure 5A). High incidence of hemizygous deletions (hemidel) and copy-neutral loss of heterozygosity (CN-LOH) events in *LIG1* characterized low-grade glioma (LGG), OV, and lung adenocarcinoma (LUAD) among others, while homozygous deletions (homodel) and deleterious single nucleotide variants (SNVs) were detected in a small fraction of several tumor types (Figure 5A). We selected LUAD, BRCA, and colon adenocarcinoma (COAD) in vitro models together with an additional PCa cell line (LNCaP) to test the effect of *LIG1* loss in combination with PARPi treatment. A549 (LUAD), MDA-MB-231 (BRCA), HCT116 (COAD), and LNCaP (PRAD) cells were transduced to KO *LIG1* and subsequently treated with OLA or TALA. Consistent with our previous results in 22Rv1, the administration of PARPi significantly decreased *LIG1*-KO cell proliferation in all tumor models, indicating that this SL interaction is conserved in different tumor types (corrected *P* values for sg*LIG1* [1] and [2] treated with PARPi <0.05) (Figure 5, B and C, and Supplemental Figure 5, B and C).

Collectively, these findings indicate that the *LIG1* and *PARP* SL interaction could be exploited for the treatment of multiple tumor types, beyond PCa.

### Combined treatment with LIG1 and PARP inhibitors selectively reduces cancer cell proliferation.

**Figure 6:**
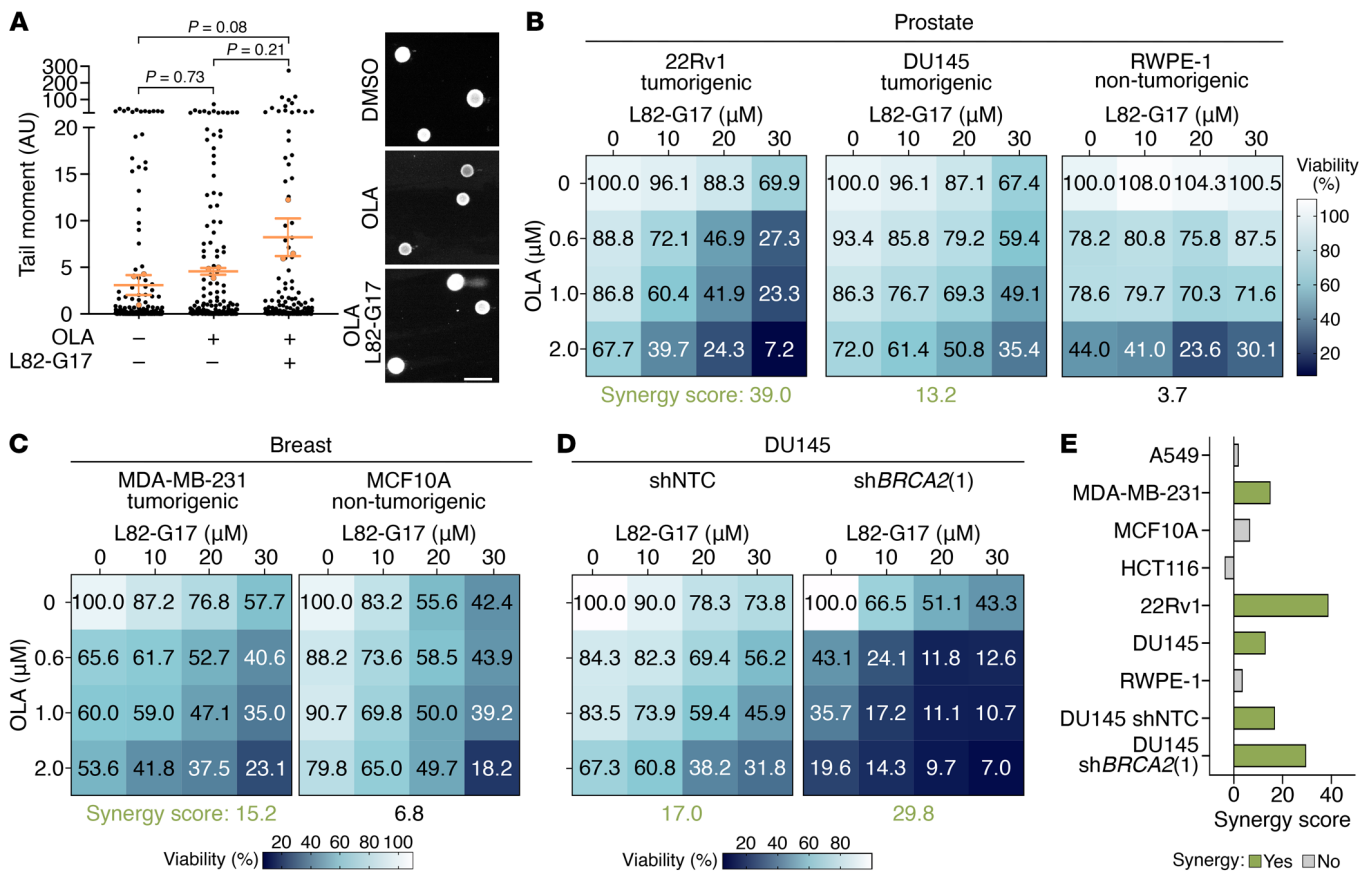
Combined pharmacological inhibition of LIG1 and PARP reduces the viability of tumor cells. (**A**) Comet tail moment measured by alkaline comet assay in 22Rv1 treated with DMSO, 2 μM OLA and 40 μM L82-G17 for 3 days. The orange dots and bars indicate the mean value of each replicate and the mean ± SEM of the 3 experiments, respectively. *P* values were determined using 1-way ANOVA and Tukey’s multiple-comparisons test. Images are representative of the comet assays. Scale bar: 100 μm. (**B**–**D**) Matrices of cell viability quantifications (crystal violet assays for **B** and **C**; CCK8 for **D**) in the indicated cell lines treated with OLA and L82-G17 for 8–17 days. Data are presented as mean (*n* = 2 biological replicates for 22Rv1 and RWPE-1, *n* = 3 biological replicates for the other cell lines). Synergy scores were calculated by using the HSA model. (**E**) Overview of the synergy scores from the cell viability experiments with L82-G17 and OLA treatment.

We sought additional approaches to leverage *LIG1* and *PARP* SL interaction and possibly extend its therapeutic applicability to models and patients with functional LIG1. We searched for LIG1-specific inhibitors and tested whether LIG1 and PARP combined pharmacological inhibition is effective in PCa and other tumor models. L82-G17, a recently developed LIG1 inhibitor, with low activity on other DNA ligases (47), was used in combination with OLA. Notably, analysis of DNA damage through alkaline comet assay highlighted an increase in the tail moment of 22Rv1 cells treated with both inhibitors compared with untreated cells, indicating that the combination of the 2 compounds induces DNA breaks in PCa cells (Figure 6A). Consistently, immunoblot analyses of γH2AX levels showed that treatment with L82-G17 and/or OLA specifically promotes DNA damage in 22Rv1 cells, while no differences were observed in nontumorigenic RWPE-1 cells (Supplemental Figure 6A).

Importantly, the combination of LIG1 and PARP inhibitors significantly reduced the survival of PCa and BRCA cells (synergy scores >10) (Figure 6, B, C, and E, and Supplemental Figure 6, B and C). In contrast, this treatment had no effect on the proliferation of prostate and breast nontumorigenic cells (RWPE-1 and MCF10A, respectively), nor did it influence the A549 and HCT116 cell lines (Figure 6, B, C, and E, and Supplemental Figure 6, B–D). These findings indicate that the concomitant pharmacological inhibition of LIG1 and PARP might represent a promising therapeutic strategy against certain cancer types.

Next, we assessed the efficacy of combining L82-G17 with OLA in BRCA2-depleted cells. DU145 cells were transduced with Dox-inducible lentiviral vectors expressing 2 shRNAs targeting *BRCA2* (sh*BRCA2*), alongside an shRNA non-targeting control (shNTC). Immunoblot analysis showed a significant reduction in BRCA2 expression with sh*BRCA2*(1), while sh*BRCA2*(2) resulted in only a modest decrease in protein levels (Supplemental Figure 6E). Therefore, only DU145-sh*BRCA2*(1) and shNTC cells were treated with Dox and increasing concentrations of L82-G17 and/or OLA for 10 days. Consistent with previous findings, survival assays in DU145-shNTC cells showed synergy between LIG1 and PARP inhibition and DU145-sh*BRCA2*(1) cells demonstrated decreased cell survival with single OLA treatment (Figure 6, D and E). Interestingly, the cytotoxic effect of OLA in DU145-sh*BRCA2*(1) cells was even more pronounced when L82-G17 was added (Figure 6, D and E), indicating that LIG1 inhibition increases the sensitivity of BRCA2-depleted cells to PARPi.

Tumors that show molecular characteristics of *BRCA*-mutant cancers (i.e., with BRCAness), in some instances, also respond to similar therapeutic strategies (48). Recently, the combination of ENZA and PARPi has demonstrated increased antitumor activity in clinical trials compared with AR inhibition alone (14, 20–22). Preclinical studies suggest this synergistic approach may partly relate to the effect of AR activity on the DNA damage response (49–52). Therefore, we tested whether ENZA enhances PARPi sensitivity in LIG1-deficient cells. We confirmed that concomitant administration of ENZA and PARPi increased apoptosis compared with single treatment in ENZA-sensitive LNCaP cells, while no such effect was observed in ENZA-resistant 22Rv1 cells (Supplemental Figure 6F). The KO of *LIG1* did not further increase the percentage of apoptotic cells in either cell line (Supplemental Figure 6F).

Altogether, this suggests that concurrent inhibition of LIG1 and PARP might be a promising therapeutic strategy for PCa and BRCA, especially in tumors with *BRCA2* deficiency.

### AZD5305 demonstrated antitumor activity in LIG1-KO PCa xenograft mouse models.

**Figure 7:**
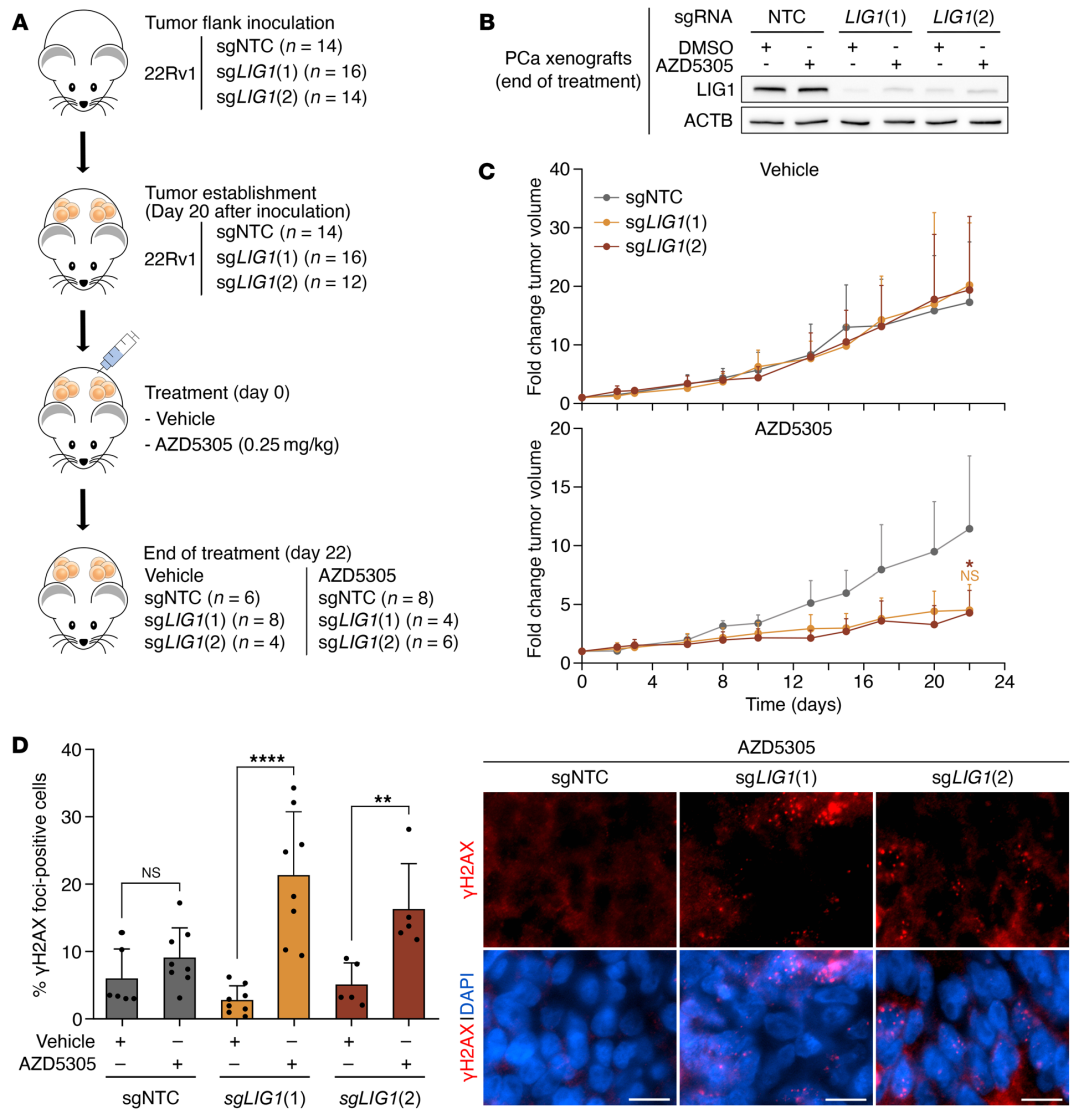
*LIG1* loss combined with PARPi treatment reduces tumor growth of PCa xenograft mouse models. (**A**) Schematic diagram of the in vivo experiments. (**B**) Immunoblot analysis of LIG1 and ACTB (used as loading control) in 6 exemplary xenograft tumor samples collected after the in vivo experiments described in **A**. (**C**) Scatter plots of the tumor volume measured during treatment with vehicle or AZD5305. Data are presented as mean + SD. (**D**) Percentage of cells with 5 or more γH2AX foci measured by immunofluorescence in FFPE xenograft tumor samples collected after the in vivo experiments described in **A**. Images are representative of 3 FFPE xenografts tumor sections stained for γH2AX (red) and nucleus (DAPI, blue). Scale bars: 12 μm. Data are presented as mean ± SD. *P* values were determined using 2-tailed unpaired *t* test. *P ≤ 0.05; **P ≤ 0.01; ****P ≤ 0.0001.

Multiple clinical trials are currently testing an additional PARPi, AZD5305, to improve the clinical efficacy and widen the therapeutic window for PCa patients. Indeed, preclinical data has demonstrated that AZD5305 is highly selective for PARP1 allowing the administration of higher doses with an improved tolerability profile (53). Considering the therapeutic potential of this novel PARPi, we tested whether AZD5305 is effective on PCa cells characterized by *LIG1* loss. Survival analyses showed that AZD5305 negatively affected *LIG1*-KO 22Rv1 cell survival (corrected *P* values for sg*LIG1* treated with 5–1000 nM AZD5305 <0.01), whereas it had mild effects on control cells (Supplemental Figure 7A). In addition, we performed in vivo experiments on PCa xenograft mouse models (Figure 7A). 22Rv1 control (sgNTC) and *LIG1*-KO (sg*LIG1*[1] and sg*LIG1*[2]) cells were injected into both flanks of each mouse and tumor growth was monitored. Once tumors were established, mice were treated with AZD5305 (0.25 mg/kg) or vehicle for approximately 3 weeks, during which tumor volumes were measured regularly. Treatment with AZD5305 showed no effects on the weight of the mice, confirming the low toxicity of the compound (Supplemental Figure 7B). In line with the in vitro results, AZD5305 treatment strongly impaired the growth of tumors characterized by *LIG1*-KO compared with control and untreated models (*t* test *P* value for sg*LIG1*[1] treated with AZD5305 = NS; *P* value for sg*LIG1*[2] treated with AZD5305 =0.046) (Figure 7, B and C). Moreover, we detected an increase in the percentages of γH2AX foci–positive cells in *LIG1*-KO samples compared with control samples (*t* test *P* values for sg*LIG1*[1] and *sgLIG1*[2] treated with AZD5305 ≤ 0.01), while no difference in RAD51 foci formation was observed, indicating DNA damage induction and antitumor activity in vivo despite maintaining functional HRR capability (Figure 7D and Supplemental Figure 7C). These in vivo results support the potential of *LIG1* loss as a predictive biomarker for CRPC patient treatment.

## Discussion

Despite the compelling results obtained with PARPi for CRPC treatment, several barriers still hinder its efficacy and applicability, including the low frequency of DRG aberrations and the high variability in treatment response. Preclinical studies have been focused on improving patient stratification and treatment; however, more specific biomarkers are still required (23, 54–57).

In this context, we performed multiple CRISPR/Cas9 screens in PCa cell lines treated with PARPi (OLA and TALA) and using a custom sgRNA library that targets an extended list of DRG (including non-HRR genes). The results of the screens highlighted several genes (*CHD1L*, *BRCA1*, *MUS81*, *RNASEH2A*, and *XRCC1*) previously associated with PARPi sensitivity (1, 25, 26, 31–34) and identified 19 DRG that potentially represent novel PCa vulnerabilities. Moreover, although the setup was not ideal for identifying genes associated with resistance to PARPi treatment, they hint at potential candidates that could warrant further investigations. Among the hits nominated for sensitization to PARPi, validation experiments confirmed the SL interaction between *LIG1*, *EME1*, and *FAAP24* losses and PARPi in PCa cells, and analyses of genomic and clinical data led to the selection of *LIG1* as the most promising hit associated with PARPi sensitivity.

The SL between *LIG1* and *PARP* has been already identified in different CRISPR/Cas9 screens (23, 45, 46, 58), but it has not been extensively studied. While this manuscript was under revision, Bhandari et al. showed that PARP1 and PARP2 have redundant roles in supporting the viability of Lig1-deficient cells and that simultaneous inhibition of both PARPs is necessary to kill murine lymphoma cells lacking Lig1 (59). Since OLA targets both PARP1 and 2, we did not examine the individual contribution of PARP1 and PARP2 in maintaining the viability of LIG1 null PCa cells. Yet, the highly specific PARP1 inhibitor AZD5305 used in our in vitro and in vivo experiments reduced the survival of LIG1-depleted 22Rv1 cells, indicating that PARP1 is the key player in the SL interaction with LIG1 in PCa. We further investigated the mechanism underlying this interaction showing increased PAR on chromatin upon depletion of LIG1 and, coupled with PARP inhibition, induction of DNA damage, γH2AX in S and G_2_/M phase cells, and phosphorylation of ATM and CHK1. Moreover, we observed no difference in the activity of the NHEJ and HRR pathways. Together with the observation of unprocessed replication gaps as determinant of *BRCA1*/*2* and *PARP* SL (60), these results support the hypothesis that the accumulation of extensive DNA damage caused by the combination of *LIG1* loss and PARPi treatment is generated by incorrect processing of Okazaki fragments. LIG1, indeed, is a DNA ligase that seals DNA nicks as the last step of several DNA repair pathways and during the processing of Okazaki fragments (35, 61, 62). When LIG1 is inactive, incompletely processed DNA fragments are recognized and bound by PARP1/2 and processed through the XRCC1/LIG3 single-strand break repair pathway (35, 63, 64). Thus, we suggest that concomitant PARP inhibition and *LIG1* loss disrupt Okazaki fragment processing and, as a consequence, lead to the accumulation of single-strand DNA gaps and DNA double-strand breaks, genomic instability, and ultimately, cell death.

The clinical relevance of the SL interaction between *LIG1* and *PARP*, was confirmed by testing the combination of *LIG1* loss and PARP inhibition across diverse preclinical tumor models, including a PCa xenograft mouse model. Of note, our results confirmed that the SL we identified is conserved also in other tumor types, beyond PCa. These findings, together with previously published data (45, 46) and our pan-cancer genomic analyses of *LIG1* aberrations, provide evidence for the use of PARPi as a treatment approach for a spectrum of tumors (e.g., PCa, LUAD, BRCA, COAD, and potentially OV) characterized by *LIG1* defects.

Further expanding the potential applicability of *LIG1* and *PARP* SL, we demonstrated the efficacy of their combined pharmacological inhibition. Notably, multiple clinical and preclinical studies demonstrated that therapeutic approaches based on drug combinations are a valuable alternative to monotherapy, especially in overcoming drug resistance (20–22, 65, 66). Our results suggest that LIG1-specific inhibitors could expand the opportunities for PCa and BRCA patient treatment, whereas other tumor types, such as LUAD and COAD, seem to be less responsive to the combined pharmacological inhibition of LIG1 and PARP. This discrepancy might be attributed to variations in LIG1 activity or insufficient LIG1 inhibition in these tumors. The development of novel LIG1 inhibitors suitable for in vivo studies (47) might help to address this discrepancy and is essential to fully evaluate the translational potential of this therapeutic strategy.

Overall, our work provides the rationale to include *LIG1* in the panel of biomarkers for PARPi-based therapy and gives initial evidence for a drug combination-based approach that may either improve treatment response or expand the patient population beyond those with *LIG1* loss-of-function aberrations. Furthermore, our findings support the potential of leveraging low-frequency aberrations in DRG, beyond HRR genes. Indeed, when considering the incidence of PCa, the identification of DRG with low-frequency mutations, as observed in the case of *LIG1*, remains noteworthy and relevant.

*LIG1* joins the growing list of non-HRR DRG, including *RNASEH2B*, *CHD1L*, and *FEN1* (23–26), previously identified as PARPi sensitizers. Collectively, this opens new research avenues based on studying how DRG not directly involved in HRR may contribute to PARPi sensitivity and supports the investigation of additional biomarkers to further expand the patient population that might benefit from this treatment. Extending the genomic profiling of samples from PARPi clinical trials with an unselected population of patients to assess the status of non-HRR DRG might provide important insights in this direction.

## Methods

### Sex as a biological variable.

Our study exclusively examined male mice since the disease modeled is only relevant in males.

### Cell culture and treatment.

LNCaP, 22Rv1, and A549 were grown in RPMI 1640 medium (Gibco, Thermo Fisher Scientific) supplemented with 10% fetal bovine serum (FBS) (MilliporeSigma), 1% l-glutamine, and 1% penicillin-streptomycin (p/s). HCT116 were grown in McCoy’s 5A medium (Gibco, Thermo Fisher Scientific) supplemented with 10% FBS (MilliporeSigma) and 1% p/s. HEK 293T, DU145 and MDA-MB-231 were grown in DMEM medium (Gibco, Thermo Fisher Scientific) supplemented with 10% FBS (MilliporeSigma), 1% l-glutamine, and 1% p/s. RWPE-1 were grown in Keratinocyte-SFM medium (Gibco, Thermo Fisher Scientific) supplemented with 5 ng/ml human recombinant epidermal growth factor (EGF), 0.05 mg/ml bovine pituitary extract, and 1% p/s. MCF10A were grown in DMEM/F-12 (Gibco, Thermo Fisher Scientific) supplemented with 5% horse serum (Gibco, Thermo Fisher Scientific), 20 ng/ml human EGF (Gibco, Thermo Fisher Scientific), 0.5 μg/ml hydrocortisone (Voden), 10 μg/ml insulin (Santa Cruz Biotechnology), and 1% p/s. Cell lines were purchased from the ATCC and/or authenticated using the ATCC STR profile as a reference. All cells were regularly tested and negative for mycoplasma contamination. PCa cell lines were profiled by targeted sequencing for the genes of interest, using the PCF_SELECT assay (67).

### Generation of sgRNA or shRNA-expressing cell lines.

sgRNAs or shRNAs targeting the gene of interest (Supplemental Table 8) were cloned into LentiCRISPR_opt_puro (modified from Addgene 70662) or pLKO_TetON_puro (Addgene 21915) vector, respectively. Lentiviral vectors were generated using psPAX2 (Addgene 12260) and pCMV-VSV-G (Addgene 8454) with polyethylenimine (PEI) transfection reagent in HEK293T cells. For transduction, cells were incubated for 24 hours with the lentiviral supernatants in the presence of 8 μg/ml polybrene (Santa Cruz Biotechnology) and then selected with 2 μg/ml puromycin (InvivoGen). Knockout efficiency was determined by immunoblot or TIDE analysis (68) while knockdown efficiency was verified by immunoblot after 2 days of treatment with ethanol (used as control) or Dox (1 μg/ml).

### Immunoblot.

To isolate proteins, cells were washed once with 1× PBS and then lysed in RIPA buffer (0.05M HEPES pH 7.9, 0.14M NaCl, 0.001M EDTA, 1% Triton X-100, sodium deoxycholate 0.1%, SDS 0.1%) supplemented with proteinase and phosphatase inhibitor cocktails (Merck). Protein concentration was determined by performing bicinchoninic acid (BCA) assay. Protein samples and the PageRuler Pre-Stained Protein Ladder (Thermo Fisher) were run on 4.5%–12% or 7% Bolt Bis-Tris Plus gels (Thermo Fisher) and then transferred to PVDF membranes (GE Healthcare Life Sciences) using the Bis-Tris buffer system. Membranes were blocked with 5% milk or BSA diluted in TBS buffer supplemented with 0.1% Tween 20 (TBS-T) for 1 hour at room temperature. Membranes were incubated overnight with primary antibodies (Supplemental Table 9) diluted in blocking buffer (5% milk or BSA in TBS-T) and then with secondary antibodies (Supplemental Table 9) diluted in 5% milk TBS-T. Amersham ECL Prime or Select reagents (GE Healthcare Life Sciences) were used to visualize proteins at the UVITec Alliance LD2.

### Immunofluorescence.

7500 22Rv1 cells were seeded in a PhenoPlate 96-well microplates (Revvity) and maintained in culture with the appropriate medium conditions. Successively, cells were fixed by adding 4% paraformaldehyde (PFA) solution directly on the well containing the medium and incubated for 15 minutes. PFA solution was then replaced with a solution containing 3% BSA, 0.3% Triton X-100 diluted in 1× PBS, and phosphatase inhibitors (1:500, MilliporeSigma), and cells were incubated for 45 minutes at room temperature. Afterwards, a solution containing the primary antibody, 1% BSA-PBS, 0.3% Triton X-100, and phosphatase inhibitors (1:500, MilliporeSigma) was added in each well. Cells were incubated for 1 hour, washed 3 times with 1× PBS including phosphatase inhibitors (1:500, MilliporeSigma), and incubated again with the secondary antibody diluted in a solution containing 3% BSA, 0.3% Triton X-100, and phosphatase inhibitors (1:500, MilliporeSigma). Finally, after being washed 3 times with 1× PBS including phosphatase inhibitors (1:500, MilliporeSigma), nuclei were stained with Hoechst 33342 (Thermo Fisher) for 10 minutes and the wells were covered with 1× PBS. Immunofluorescence analysis was performed with ImageXpress Micro Confocal (Molecular Devices).

### Cell survival and viability assays.

Cells were seeded in 48-well plates at low density and treated as indicated for 8–17 days. Media was replaced every 2 days. For crystal violet assay, cells were fixed with formaldehyde (4%) for 10 minutes and stained with crystal violet solution (0.1%) for 30 minutes at room temperature. To calculate relative cell viability, each well was destained by adding acetic acid (10%) for 20 minutes and the absorbance was measured at 590 nm by using Varioskan LUX Multimode Microplate reader (Thermo Fisher Scientific).

For the CCK8 assay, CCK8 solution (2.5%, Dojindo) was added to each well, and cells were incubated for 1 hour in a CO_2_ incubator. Absorbance was measured at 450 nm using a Varioskan LUX Multimode Microplate reader (Thermo Fisher Scientific).

### Drug synergy analysis.

Cells were seeded in 48-well plate and treated with DMSO (as control) or 3 doses of OLA (0.6, 1, 2 μM) and L82-G17 (10, 20, 30 μM) in a matrix format. After 14 days, a crystal violet survival assay was performed, and the percentage of cell viability was calculated. Drug synergy scores were calculated based on the HSA model using the SynergyFinder3.0 web-based tool (https://synergyfinder.fimm.fi/) (69).

### CellEvent analysis.

Cells were seeded in 96-well plate (8,000 cells/well) and treated with DMSO (as control) or PARPi (OLA or TALA) for 3–5 days. After treatment, Hoechst and 1μM CellEvent caspase-3/7 detection reagent (Thermo Fisher) were added to each well, and cells were incubated for 1 hour in a CO_2_ incubator. Analyses were performed with ImageXpress Micro Confocal High-Content Imaging System (Molecular Devices).

### Flow cytometry analyses.

For annexin V/PI FACS cells were seeded in 6-well plates and treated with DMSO (as control) or PARPi for 3 days. 2 × 10^5^ cells were collected, washed twice with 1× PBS, resuspended in 100 μL of 1× binding buffer and incubated with 5μL APC annexin-V (BD Biosciences — Pharmingen, Supplemental Table 9) for 15 minutes at room temperature. After incubation, 400 μL 1× binding buffer and 5 μL PI were added, and samples were analyzed.

For cell cycle analysis coupled with γH2AX detection, the Click-iT Plus EdU Flow Cytometry Assay Kit (C10635, Thermo Fisher Scientific) was used. Cells were labeled with 10 μM EdU for 2 hours and then harvested by trypsinization, pelleted by centrifugation at 400*g* for 5 minutes, washed, and resuspended in 1ml 1× PBS. Cells were fixed in ice-cold EtOH and kept at –20°C at least overnight. The Click-iT reaction was carried out as indicated in the manufacturer’s instructions. Afterwards, cells were washed with 1% BSA-PBS and incubated with γH2AX antibody in 1% BSA-PBS for 1 hour at room temperature. After washing, Alexa Fluor 488 (Invitrogen) secondary antibody in 1% BSA-PBS was added to the cells for 30 minutes at room temperature in the dark. Finally, cells were resuspended in 1× PBS containing RNase A and PI, incubated for 30 minutes at 37°C, and analyzed.

All FACS samples were analyzed on a FACSymphony A1 Cell Analyzer (BD Biosciences) and quantified using the FlowJo Software (Version 10.9.0, BD Biosciences).

### Alkaline comet assay.

Alkaline comet assay was performed on 22Rv1 cells treated as indicated using CometAssay Kit (R&D Systems, catalog 4250-050-K), following the manufacturer’s instructions. Tail moment was measured using CometScore 2.0 software.

### HRR-EGFP assay.

HEK293T-EGFP reporter cells were provided by Cereseto’s laboratory and transduced first with the LentiCRISPR_opt encoding sgNTC, sg*LIG1*(1), and sg*LIG1*(2) and subsequently with LentiCRISPRv1_sgI-SceI (modified from ref. 70). Six days after transduction and 5 days after the start of the indicated treatments, cells were collected, washed with 1× PBS and resuspended in 1 ml 1× PBS. Samples were analyzed by flow cytometry using FACSymphony A1 Cell Analyzer (BD Biosciences — Pharmingen) and the percentage of EGFP-positive cells was determined using FlowJo software (version 10.9.0, BD Biosciences).

### CRISPR/Cas9 screens.

The custom sgRNA library (Merck) included 1,676 sgRNAs (4 sgRNAs/gene) that target 356 genes of interest and 63 essential genes, and 324 NTC (Supplemental Table 1). sgRNA sequences were taken for the Brunello library (71) or provided by Merck. The genes of interest comprised DRG selected from the Molecular Signatures Database, version 7.5.1 (MSigDB) (72, 73) and the gene panels used in the TOPARP-B and TRITON2 trials (15, 17). The essential genes were taken from a previous manuscript (27) and from the analysis of Dependency Map (DepMap, Broad Institute; refs. 28, 29) data in PCa cell lines for spliceosome and ribosomal genes.

For the screens, 22Rv1 and DU145 LentiCas9 (pLentiCas9_blastR, Addgene 52962) clones were isolated by limiting dilution and tested for Cas9 activity with an efficient sgRNA by TIDE analysis. Clones with an editing efficiency higher than 70% were selected for the screen. Cells were seeded (16.2 × 10^6^ 22Rv1 cells and 7.2 × 10^6^ DU145 cells) and transduced with the lentiviral custom sgRNA library at a low MOI (about 0.3). After selection with puromycin, a subset of cells corresponding to the T_0_ sample was collected (to have 500–300× coverage), while the rest were seeded to have a 300× cell coverage and treated with 0.1 μM OLA (Selleck Chemicals, catalog S1060), 3–5 nM TALA (Selleck Chemicals, catalog S7048), or DMSO (as control) for 15–18 population doublings (during which cell culture medium was replaced every 48 hours). Once the final time point was reached, cells were harvested and gDNA was isolated using QIAamp DNA Blood Mini Kit (QIAGEN). Genome-integrated sgRNA sequences were amplified by PCR using TaKaRa Ex Taq DNA Polymerase (Takara Bio Group, catalog RR001A; primers listed in Supplemental Table 8) and double-size selection with AMPure XP (Beckman Coulter) was performed to purify the PCR product of interest. Samples were sequenced using the Illumina MiSeq.

To identify putative hits, the DrugZ algorithm with default parameters was used (30). Given the custom design of the library, essential genes were excluded from count tables, and sgRNAs NTC were randomly aggregated in groups of 4 to be treated as single genes. The procedure was repeated to generate a total of *n* = 100 count tables for each cell line and drug. The DrugZ algorithm was then applied separately on each table and Norm *z* scores were computed as the average of the *n* = 100 repetitions (Supplemental Tables 2–7). The *P* values were calculated from the averaged *z* scores and corrected for multiple hypothesis testing using the method of Benjamini and Hochberg.

To nominate candidates for validation, genes with a DMSO versus T_0_ NormZ score (DMSO versus T_0_) between –1 and +1 and a NormZ score (OLA or TALA versus DMSO) lower than –1 in at least one cell line were selected and then further subselected based on the FDR (OLA or TALA versus DMSO) (<0.1) and/or the consistency of the signal in the plots comparing the sgRNA counts for each gene in DMSO- and OLA- or TALA-treated samples.

### In vivo xenograft studies.

To establish human PCa xenograft models in NMRI-Foxn1 nu/nu immunodeficient mice (supplied from Janvier-Labs), 22Rv1 cells expressing sgRNAs (sgNTC, sg*LIG1*[1], sg*LIG1*[2]) were injected into both flanks (3 × 10^6^ cells in 100 μL medium) of 4-week-old animals. Tumor growth was monitored regularly using a digital caliper, and the tumor volume was calculated using the formula (width)^2^ × length/2. After 20 days from engraftment, the 3 different groups of tumor-bearing mice (sgNTC [*n* = 7], sg*LIG1*[1] [*n* = 8], and sg*LIG1*[2] [*n* = 6]) were randomized into vehicle and treatment groups. The vehicle (H_2_O pH = 3.5) and the AZD5305 drug (0.25 mg/kg) were administrated 6 times per week by oral administration (100 μL). Mice were euthanized 22 days after treatment or earlier if tumors reached maximum ethical size. Immediately following euthanasia, a portion of the collected tumors were snap-frozen in liquid nitrogen to preserve DNA, RNA, and proteins; and another portion of the collected tumors was fixed in 10% neutral-buffered formalin to preserve the tissue structure. After fixation, the tumors were dehydrated in a series of alcohol washes and embedded in paraffin wax to obtain formalin-fixed paraffin-embedded (FFPE) blocks.

### Immunofluorescence of FFPE samples.

For target antigen retrieval, the sections underwent a heat treatment, involving microwaving at 110°C for 4 minutes in DAKO Antigen Retrieval Buffer at pH 9.0, facilitated by a T/T MEGA multifunctional Microwave Histoprocessor (Milestone). The sections were cooled down in distilled water for 30 minutes, and then subjected to permeabilization using DAKO Wash Buffer, containing Tween-20, for 5 minutes. Subsequently, a 5-minute incubation in a blocking buffer (DAKO Wash Buffer supplemented with 1% bovine serum albumin) was carried out. Primary antibodies (Supplemental Table 9) were diluted in DAKO Antibody Diluent and incubated at room temperature for 1 hour. After this step, the sections were washed for 5 minutes in DAKO Wash Buffer, followed by another 5-minute incubation in blocking buffer. Secondary antibodies (Supplemental Table 9), diluted in blocking buffer, were then incubated with the sections for 30 minutes at room temperature. The 2-step washing process was repeated, followed by a 5-minute incubation in distilled water. Dehydration was systematically performed using a series of ethanol solutions with increasing concentrations. Finally, the sections were mounted with DAPI ProLong Gold antifading reagent (Invitrogen) and stored at –20°C. Immunofluorescence images were captured utilizing an Olympus DP72 microscope and processed with CellSens Entry software. The extent of DNA damage was quantified on FFPE xenograft tumor samples by evaluating the percentage of yH2AX-positive cells relative to all DAPI-stained cells. The quantification of RAD51 foci, measuring between 0.42 and 1.15 μm in diameter, was conducted on FFPE xenograft tumor samples as described in (74) with modifications. This involved scoring the percentage of yH2AX-positive cells with 5 or more RAD51 nuclear foci. The scoring process was carried out blindly on live images using a 60× immersion oil lens. Analyses were performed on a minimum of 2 biological replicates for each xenograft model, both vehicle and AZD5305 treated.

### Genomic and transcriptomic human sample data processing.

Data from TCGA (43) and the SU2C-PCF (5) cohorts were queried for germline and somatic aberrations in selected genes. Only high-quality sample data (i.e., 4,950 across 27 different tumor types for TCGA and 399 for SU2C-PCF) amenable to allele-specific genomic analysis (SPICE pipeline; ref. 44) were considered. Specifically, allele-specific copy number calls corrected by tumor ploidy and purity (CLONETv2; ref. 75), nonsynonymous SNV and indel calls (MuTect2 [ref. 76] calls annotated with variant effect predictor (VEP) [ref. 77]) were used together with transcriptomic data (i.e., recount2 counts [ref. 78] and normalized fragments per kilobase of transcript per million mapped reads (FPKM) for TCGA and SU2C, respectively). For the PCa data sets (297 samples from the TCGA-PRAD cohort and 399 samples from SU2C-PCF), germline mutation annotation from previous analyses (6) was used and only germline events with a high allelic fraction (AF ≥ 0.35) and with a likely pathogenic effect (consequence field different from “intron_variant”, “synonymous_variant”, “inframe_deletion”, “inframe_insertion” and annotated by Clinvar as “pathogenic” or “risk_factor”) were considered.

### Tumor stage analyses.

The association between the concomitant expression of the candidate genes (*LIG1*, *EME1*, *FAAP24*) and *PARP1* with the tumor stage was performed leveraging the TCGA-PRAD expression data downloaded from recount3 (79). For each gene pair (candidate gene versus *PARP1*), expression levels were discretized into “low” and “high” based on the median expression value of each gene. Pearson’s χ^2^ test was used to assess the statistical significance for intergroup differences. Results with *P* ≤ 0.05 were considered statistically significant.

### Statistics.

Two-way ANOVA test, followed by Bonferroni’s correction, was used to assess the statistical significance of intergroup differences in the following experiments: crystal violet survival assay, CCK8 cell viability assay (Figure 3A), immunofluorescence, and CellEvent analyses. Two-tailed *t* test was used to assess the statistical significance of intergroup differences in the in vivo experiments and immunofluorescence analyses. One-way ANOVA test, followed by Tukey’s correction, was used to assess the statistical significance of intergroup differences in the comet assay and CCK8 cell viability assay (Figure 5C). One-sided Wilcoxon’s signed-rank test was used to assess the statistical significance of intergroup differences in the analysis of *LIG1* expression in the pan-cancer TCGA dataset. Pearson’s χ^2^ test was used to evaluate the statistical significance of intergroup differences in the analysis of the association between gene expression and TNM tumor stage. Results with *P* ≤ 0.05 were considered statistically significant. Other statistics are included in the specific paragraphs.

### Study approval.

All experimental protocols using mouse models at Vall d’Hebron Institute of Oncology (VHIO) were approved and monitored by the Vall d’Hebron Institute of Research Animal Experimentation Ethics Committee (CEEA; registration number 68/20) in accordance with relevant local and EU regulations. All mice were maintained at the animal facility of the VHIO in strict adherence to Spanish and European Union regulations; the project was approved by the local ethics committee. The experiment was performed respecting all ethical requirements and protocols, including the new Directive (Directive 2010/63/EU) which revises Directive 86/609/EEC on protection of animals used for scientific purposes. Mice were maintained under specific pathogen–free conditions.

### Data availability.

Values for all data points in graphs are reported in the Supporting Data Values file. CRISPR/Cas9 screen data are available in the Supplemental Tables.

## Author contributions

GF, FL, and FD designed the research. GF and FL performed the research. FO, GDA, TC, and DP performed the in silico analyses. UG ran the comet assays and UG and FDADF discussed the results. ASC conducted the in vivo experiments and ASC and JM discussed the results. FRS and HK provided DRG domain knowledge. GF, FL, and FD wrote the manuscript, and all the authors edited and approved the final manuscript.

## Supplementary Material

Supplemental data

Unedited blot and gel images

Supplemental tables 1-9

Supporting data values

## Version 1 12/24/2024

In-Press Preview

## Version 2 02/17/2025

Electronic publication

## References

[1] Farmer H (2005). Targeting the DNA repair defect in BRCA mutant cells as a therapeutic strategy. Nature.

[2] Bryant HE (2005). Specific killing of BRCA2-deficient tumours with inhibitors of poly(ADP-ribose) polymerase. Nature.

[3] Abeshouse A (2015). The molecular taxonomy of primary prostate cancer. Cell.

[4] Robinson D (2015). Integrative clinical genomics of advanced prostate cancer. Cell.

[5] Abida W (2019). Genomic correlates of clinical outcome in advanced prostate cancer. Proc Natl Acad Sci U S A.

[6] Armenia J (2018). The long tail of oncogenic drivers in prostate cancer. Nat Genet.

[7] Quigley DA (2018). Genomic hallmarks and structural variation in metastatic prostate cancer. Cell.

[8] Pritchard CC (2016). Inherited DNA-repair gene mutations in men with metastatic prostate cancer. N Engl J Med.

[9] Abida W (2017). Prospective genomic profiling of prostate cancer across disease states reveals germline and somatic alterations that may affect clinical decision making. JCO Precis Oncol.

[10] Castro E (2015). Effect of BRCA mutations on metastatic relapse and cause-specific survival after radical treatment for localised prostate cancer. Eur Urol.

[11] Castro E (2019). PROREPAIR-B: a prospective cohort study of the impact of germline DNA repair mutations on the outcomes of patients with metastatic castration-resistant prostate cancer. J Clin Oncol.

[12] Castro E (2013). Germline BRCA mutations are associated with higher risk of nodal involvement, distant metastasis, and poor survival outcomes in prostate cancer. J Clin Oncol.

[13] Darst BF (2021). Germline sequencing DNA repair genes in 5545 men with aggressive and nonaggressive prostate cancer. J Natl Cancer Inst.

[14] Mateo J (2015). DNA-repair defects and olaparib in metastatic prostate cancer. N Engl J Med.

[15] Mateo J (2020). Olaparib in patients with metastatic castration-resistant prostate cancer with DNA repair gene aberrations (TOPARP-B): a multicentre, open-label, randomised, phase 2 trial. Lancet Oncol.

[16] de Bono J (2020). Olaparib for metastatic castration-resistant prostate cancer. N Engl J Med.

[17] Abida W (2020). Rucaparib in men with metastatic castration-resistant prostate cancer harboring a *BRCA1* or *BRCA2* gene alteration. J Clin Oncol.

[18] Fizazi K (2023). Rucaparib or physician’s choice in metastatic prostate cancer. N Engl J Med.

[19] Abida W (2020). Non-BRCA DNA damage repair gene alterations and response to the PARP inhibitor rucaparib in metastatic castration-resistant prostate cancer: analysis from the phase II TRITON2 study. Clin Cancer Res.

[20] Saad F (2023). Olaparib plus abiraterone versus placebo plus abiraterone in metastatic castration-resistant prostate cancer (PROpel): final prespecified overall survival results of a randomised, double-blind, phase 3 trial. Lancet Oncol.

[21] Clarke NW (2022). Abiraterone and olaparib for metastatic castration-resistant prostate cancer. NEJM Evid.

[22] Agarwal N (2023). Talazoparib plus enzalutamide in men with first-line metastatic castration-resistant prostate cancer (TALAPRO-2): a randomised, placebo-controlled, phase 3 trial. Lancet.

[23] Tsujino T (2023). CRISPR screens reveal genetic determinants of PARP inhibitor sensitivity and resistance in prostate cancer. Nat Commun.

[24] Vaitsiankova A (2022). PARP inhibition impedes the maturation of nascent DNA strands during DNA replication. Nat Struct Mol Biol.

[25] Zimmermann M (2018). CRISPR screens identify genomic ribonucleotides as a source of PARP-trapping lesions. Nature.

[26] Hewitt G (2021). Defective ALC1 nucleosome remodeling confers PARPi sensitization and synthetic lethality with HRD. Mol Cell.

[27] Hart T (2015). High-Resolution CRISPR screens reveal fitness genes and genotype-specific cancer liabilities. Cell.

[28] Meyers RM (2017). Computational correction of copy number effect improves specificity of CRISPR-Cas9 essentiality screens in cancer cells. Nat Genet.

[29] Dempster JM (2021). Chronos: a cell population dynamics model of CRISPR experiments that improves inference of gene fitness effects. Genome Biol.

[30] Colic M (2019). Identifying chemogenetic interactions from CRISPR screens with drugZ. Genome Med.

[31] Ali R (2020). PARP1 blockade is synthetically lethal in XRCC1 deficient sporadic epithelial ovarian cancers. Cancer Lett.

[32] Juhász S (2020). The chromatin remodeler ALC1 underlies resistance to PARP inhibitor treatment. Sci Adv.

[33] Wang T (2022). MUS81 inhibition enhances the anticancer efficacy of talazoparib by impairing ATR/CHK1 signaling pathway in gastric cancer. Front Oncol.

[34] Zhong A (2018). Inhibition of MUS81 improves the chemical sensitivity of olaparib by regulating MCM2 in epithelial ovarian cancer. Oncol Rep.

[36] Anurag M (2022). Proteogenomic markers of chemotherapy resistance and response in triple-negative breast cancer. Cancer Discov.

[37] Abraham J (2003). Eme1 is involved in DNA damage processing and maintenance of genomic stability in mammalian cells. EMBO J.

[38] Dehé PM, Gaillard PHL (2017). Control of structure-specific endonucleases to maintain genome stability. Nat Rev Mol Cell Biol.

[39] Matos J, West SC (2014). Holliday junction resolution: regulation in space and time. DNA Repair (Amst).

[40] Osman F, Whitby MC (2007). Exploring the roles of Mus81-Eme1/Mms4 at perturbed replication forks. DNA Repair (Amst).

[41] Ciccia A (2007). Identification of FAAP24, a Fanconi anemia core complex protein that interacts with FANCM. Mol Cell.

[42] Collis SJ (2008). FANCM and FAAP24 function in ATR-mediated checkpoint signaling independently of the Fanconi anemia core complex. Mol Cell.

[43] Liu J (2018). An integrated TCGA pan-cancer clinical data resource to drive high-quality survival outcome analytics. Cell.

[44] Ciani Y (2022). Allele-specific genomic data elucidate the role of somatic gain and copy-number neutral loss of heterozygosity in cancer. Cell Syst.

[45] Jamal K (2022). Drug-gene interaction screens coupled to tumor data analyses identify the most clinically relevant cancer vulnerabilities driving sensitivity to PARP inhibition. Cancer Res Commun.

[46] DeWeirdt PC (2020). Genetic screens in isogenic mammalian cell lines without single cell cloning. Nat Commun.

[47] Howes TRL (2017). Structure-activity relationships among DNA ligase inhibitors: Characterization of a selective uncompetitive DNA ligase I inhibitor. DNA Repair (amst).

[48] Lord CJ, Ashworth A (2016). BRCAness revisited. Nat Rev Cancer.

[49] Li L (2017). Androgen receptor inhibitor-induced “BRCAness” and PARP inhibition are synthetically lethal for castration-resistant prostate cancer. Sci Signal.

[50] Asim M (2017). Synthetic lethality between androgen receptor signalling and the PARP pathway in prostate cancer. Nat Commun.

[51] Polkinghorn WR (2013). Androgen receptor signaling regulates DNA repair in prostate cancers. Cancer Discov.

[52] Goodwin JF (2013). A hormone-DNA repair circuit governs the response to genotoxic insult. Cancer Discov.

[53] Illuzzi G (2022). Preclinical characterization of AZD5305, a next-generation, highly selective PARP1 inhibitor and trapper. Clin Cancer Res.

[54] Rafiei S (2020). *ATM* loss confers greater sensitivity to ATR inhibition than PARP inhibition in prostate cancer. Cancer Res.

[55] Cahuzac M (2022). Development of olaparib-resistance prostate cancer cell lines to identify mechanisms associated with acquired resistance. Cancers (Basel).

[56] Ipsen MB (2022). A genome-wide CRISPR-Cas9 knockout screen identifies novel PARP inhibitor resistance genes in prostate cancer. Oncogene.

[57] Miao C (2022). *RB1* loss overrides PARP inhibitor sensitivity driven by *RNASEH2B* loss in prostate cancer. Sci Adv.

[58] Serrano-Benitez A (2023). Unrepaired base excision repair intermediates in template DNA strands trigger replication fork collapse and PARP inhibitor sensitivity. EMBO J.

[59] Bhandari SK (2024). Redundant but essential functions of PARP1 and PARP2 in DNA ligase I-independent DNA replication. Nucleic Acids Res.

[60] Cong K (2021). Replication gaps are a key determinant of PARP inhibitor synthetic lethality with BRCA deficiency. Mol Cell.

[61] Pascucci B (1999). Long patch base excision repair with purified human proteins. DNA ligase I as patch size mediator for DNA polymerases delta and epsilon. J Biol Chem.

[62] Paul-Konietzko K (2015). DNA Ligases I and III support nucleotide excision repair in DT40 cells with similar efficiency. Photochem Photobiol.

[63] Kumamoto S (2021). HPF1-dependent PARP activation promotes LIG3-XRCC1-mediated backup pathway of Okazaki fragment ligation. Nucleic Acids Res.

[64] Arakawa H, Iliakis G (2015). Alternative okazaki fragment ligation pathway by DNA ligase III. Genes (Basel).

[65] Chi KN (2023). Niraparib plus abiraterone acetate with prednisone in patients with metastatic castration-resistant prostate cancer and homologous recombination repair gene alterations: second interim analysis of the randomized phase III MAGNITUDE trial. Ann Oncol.

[66] Neeb A (2021). Advanced prostate cancer with ATM Loss: PARP and ATR inhibitors. Eur Urol.

[67] Orlando F (2022). Allele-informed copy number evaluation of plasma DNA samples from metastatic prostate cancer patients: the PCF_SELECT consortium assay. NAR Cancer.

[68] Brinkman EK (2014). Easy quantitative assessment of genome editing by sequence trace decomposition. Nucleic Acids Res.

[69] Ianevski A (2022). SynergyFinder 3.0: an interactive analysis and consensus interpretation of multi-drug synergies across multiple samples. Nucleic Acids Res.

[70] Moynahan ME (2001). BRCA2 is required for homology-directed repair of chromosomal breaks. Mol Cell.

[71] Doench JG (2016). Optimized sgRNA design to maximize activity and minimize off-target effects of CRISPR-Cas9. Nat Biotechnol.

[72] Subramanian A (2005). Gene set enrichment analysis: a knowledge-based approach for interpreting genome-wide expression profiles. Proc Natl Acad Sci U S A.

[73] Liberzon A (2011). Molecular signatures database (MSigDB) 3.0. Bioinformatics.

[74] Castroviejo-Bermejo M (2018). A RAD51 assay feasible in routine tumor samples calls PARP inhibitor response beyond BRCA mutation. EMBO Mol Med.

[75] Prandi D, Demichelis F (2019). Ploidy- and purity-adjusted allele-specific DNA analysis using CLONETv2. Curr Protoc Bioinformatics.

[77] McLaren W (2016). The ensembl variant effect predictor. Genome Biol.

[78] Collado-Torres L (2017). Reproducible RNA-seq analysis using recount2. Nat Biotechnol.

[79] Wilks C (2021). recount3: summaries and queries for large-scale RNA-seq expression and splicing. Genome Biol.

