# Supplementary material for: CRISPR/Cas9 screens identify LIG1 as a sensitizer of PARP inhibitors in castration-resistant prostate cancer
Source: J Clin Invest. 2024 Dec 24;135(4):e179393. doi: 10.1172/JCI179393 (PMC11827843; doi:10.1172/JCI179393)
Supplement: Supplemental data [file jci-135-179393-s162.pdf]

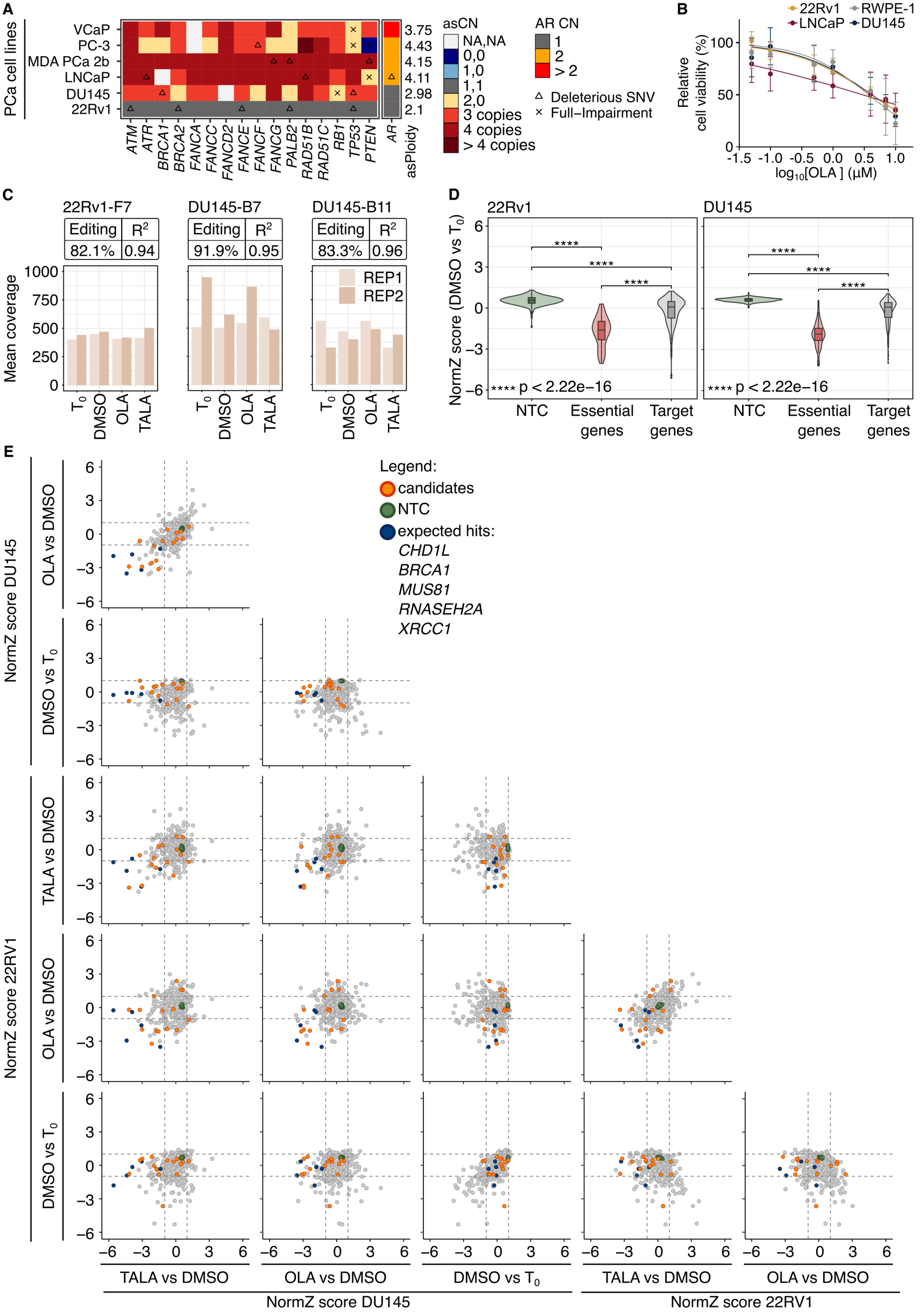
A
PCa cell lines
VCaP
PC-3
MDA PCa 2b
LNCaP
DU145
22Rv1
ATM ATR BRCA1 BRCA2 FANCA FANCC FANCD2 FANCE FANCF FANCG PALB2 RAD51B RAD51C RB1 TP53 PTEN
AR
asPloidy
3.75
4.43
4.15
4.11
2.98
2.1
asCN
NA,NA
0,0
1,0
1,1
2,0
3 copies
4 copies
> 4 copies
AR CN
1
2
> 2
Deleterious SNV
Full−Impairment
B
22Rv1
RWPE-1
LNCaP
DU145
Relative cell viability (%)
log10[OLA] (µM)
C
22Rv1-F7
DU145-B7
DU145-B11
Editing
R2
82.1%
0.94
91.9%
0.95
83.3%
0.96
Mean coverage
REP1
REP2
T0
DMSO
OLA
TALA
D
22Rv1
DU145
NormZ score (DMSO vs T0)
**** p < 2.22e−16
NTC
Essential genes
Target genes
E
Legend:
candidates
NTC
expected hits:
CHD1L
BRCA1
MUS81
RNASEH2A
XRCC1
NormZ score DU145
OLA vs DMSO
DMSO vs T0
NormZ score 22RV1
TALA vs DMSO
OLA vs DMSO
DMSO vs T0
TALA vs DMSO
OLA vs DMSO
DMSO vs T0
NormZ score DU145
TALA vs DMSO
OLA vs DMSO
NormZ score 22RV1

Supplemental Figure 1| CRISPR/Cas9 screen quality control and comparison of the results.

A Oncoprint of PCa cell lines allele-specific copy number (asCN) status of selected genes. Cell lines were profiled in-house using a prostate cancer-tailored sequencing assay (1) **B** Dose-response curve of the indicated cell lines following 6 days of treatment with OLA. Data are presented as mean + SD (n=4 biological replicates). **C** Cas9 editing efficiency and mean sequencing coverage of each screened sample. **D** Violin plot of the NormZ scores (DMSO vs T_0) of negative controls, essential genes (2–4), and target genes in the indicated cell lines. P-values were determined using the Wilcoxon signed-rank test. **E** Scatter plot matrix comparing the results of the 22Rv1 and DU145 CRISPR/Cas9 screens. Dashed lines indicate the +1 and -1 NormZ scores.

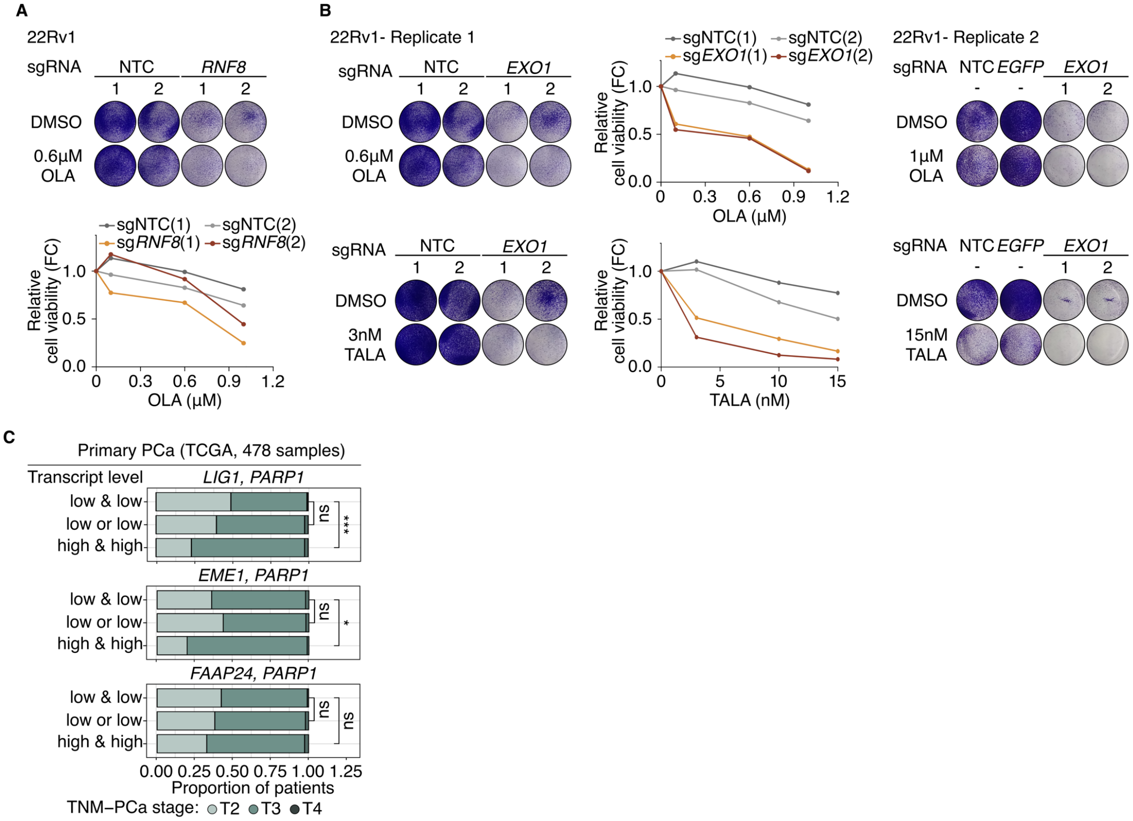

Supplemental Figure 2| Validation of *EXO1* and *RNF8* hits and analysis of the co-expression of *LIG1*, *EME1*, or *FAAP24* and *PARP1* in relation with the TNM stage in PCa samples.

A Representative images of the crystal violet assays with the corresponding quantifications. 22Rv1 cells transduced with the indicated sgRNAs were treated with DMSO (as control) or OLA at different concentrations for 13 days. **B** Representative images of the crystal violet assays (replicate 1 and 2) and quantification (replicate 1) of 22Rv1 cells transduced with the indicated sgRNAs and treated with DMSO (as control), OLA, or TALA at different concentrations for 13 days. **C** Stratification of primary PCa patients (TCGA data (5, 6)) based on TNM-tumor stage and transcript levels of *LIG1* and *PARP1*, *EME1* and *PARP1*, and *FAAP24* and *PARP1*. High and low expression stratification was defined based on the median expression value of each gene. P-values were determined using the Pearson's chi-squared test. * p-value ≤ 0.05; *** p-value ≤ 0.001. *RNF8* (panel A) and *EXO1*-OLA (replicate 1, panel B) experiments were conducted concurrently and share the same controls.

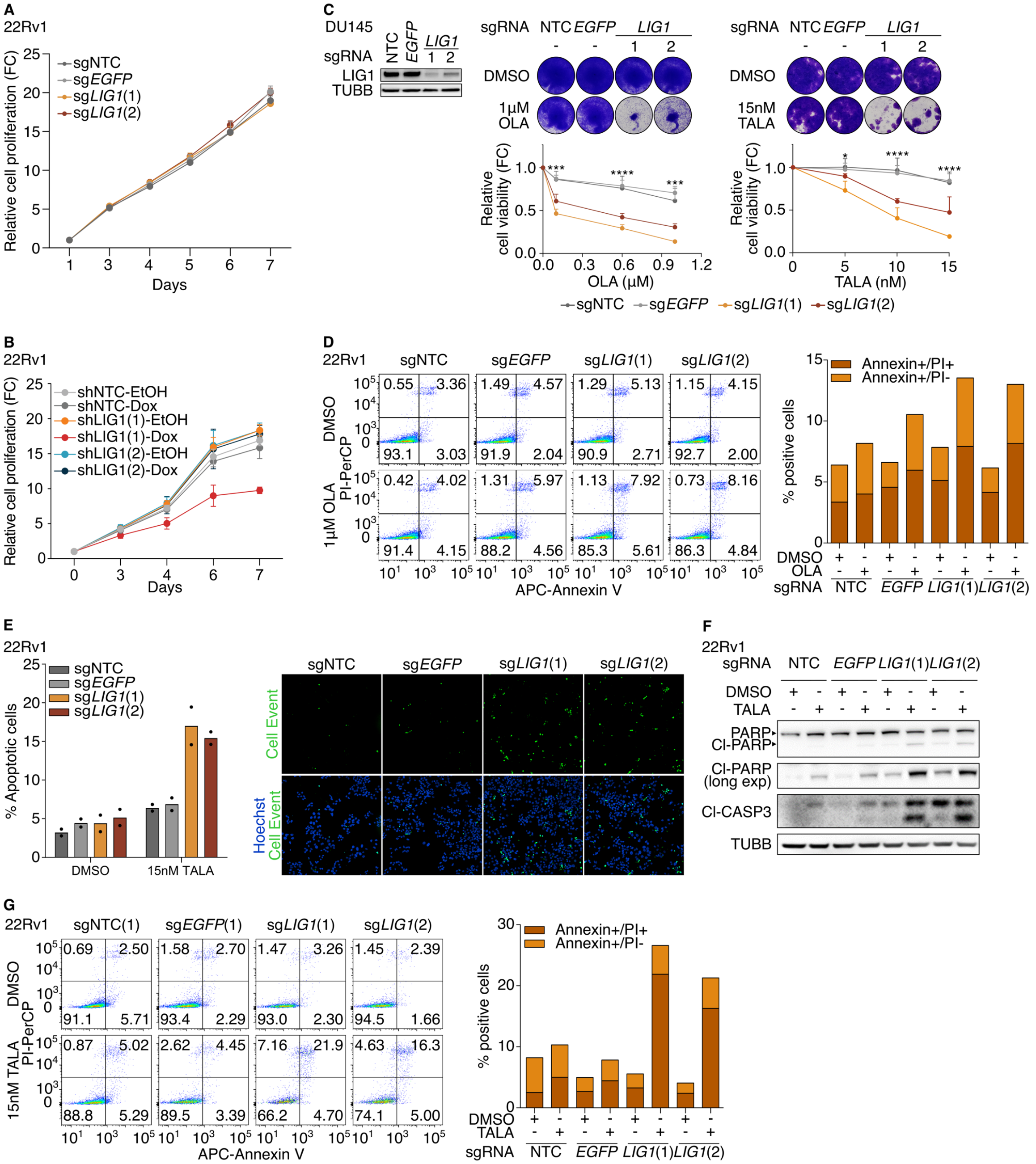

Supplemental Figure 3| *LIG1* loss combined with PARPi treatment induces apoptosis in 22Rv1 cells.

A Cell proliferation of 22Rv1 transduced with the indicated sgRNAs. Proliferation was measured with the Incucyte Live-Cell Analysis System (Sartorius) for 7 days. Data are presented as mean ± SD (n=3 biological replicates). **B** Cell proliferation of 22Rv1 transduced with the indicated shRNAs and treated ethanol (EtOH), as control, or 1 µg/ml doxycycline (Dox) for 7 days. Data are presented as mean ± SD (n=3 biological replicates). **C** Immunoblot analysis and representative images with corresponding quantification of the crystal violet assays of DU145 cells transduced with the indicated sgRNAs. Cells were treated with OLA or TALA for 11 days (DMSO was used as control). Data are presented as mean + SD (n=3 biological replicates). P-values were determined using the two-way ANOVA and Bonferroni's multiple comparisons test on control (sgNTC and sgEGFP) and sg*LIG1* samples. *** p-value ≤ 0.001; **** p-value ≤ 0.0001. **D** Representative images and bar plot of Annexin V/PI flow cytometry analysis.

22Rv1 cells transduced with the indicated sgRNAs were treated with DMSO, as control, or OLA for 3 days. **E** Percentage of apoptotic cells measured by CellEvent Caspase 3/7 assay in 22Rv1 cells transduced with the indicated sgRNA and treated with DMSO, as control, or TALA for 3 days. Images are representative of 22Rv1 cells treated with TALA. Data are presented as mean (n=2 biological replicates). **F** Immunoblot of the indicated proteins in 22Rv1 transduced and treated as in E. **G** Representative images and bar plot of Annexin V/PI flow cytometry analysis. 22Rv1 cells were transduced and treated as in E. Cl-PARP: cleaved PARP; long exp: longer exposure; Cl-CASP3: cleaved Caspase-3.

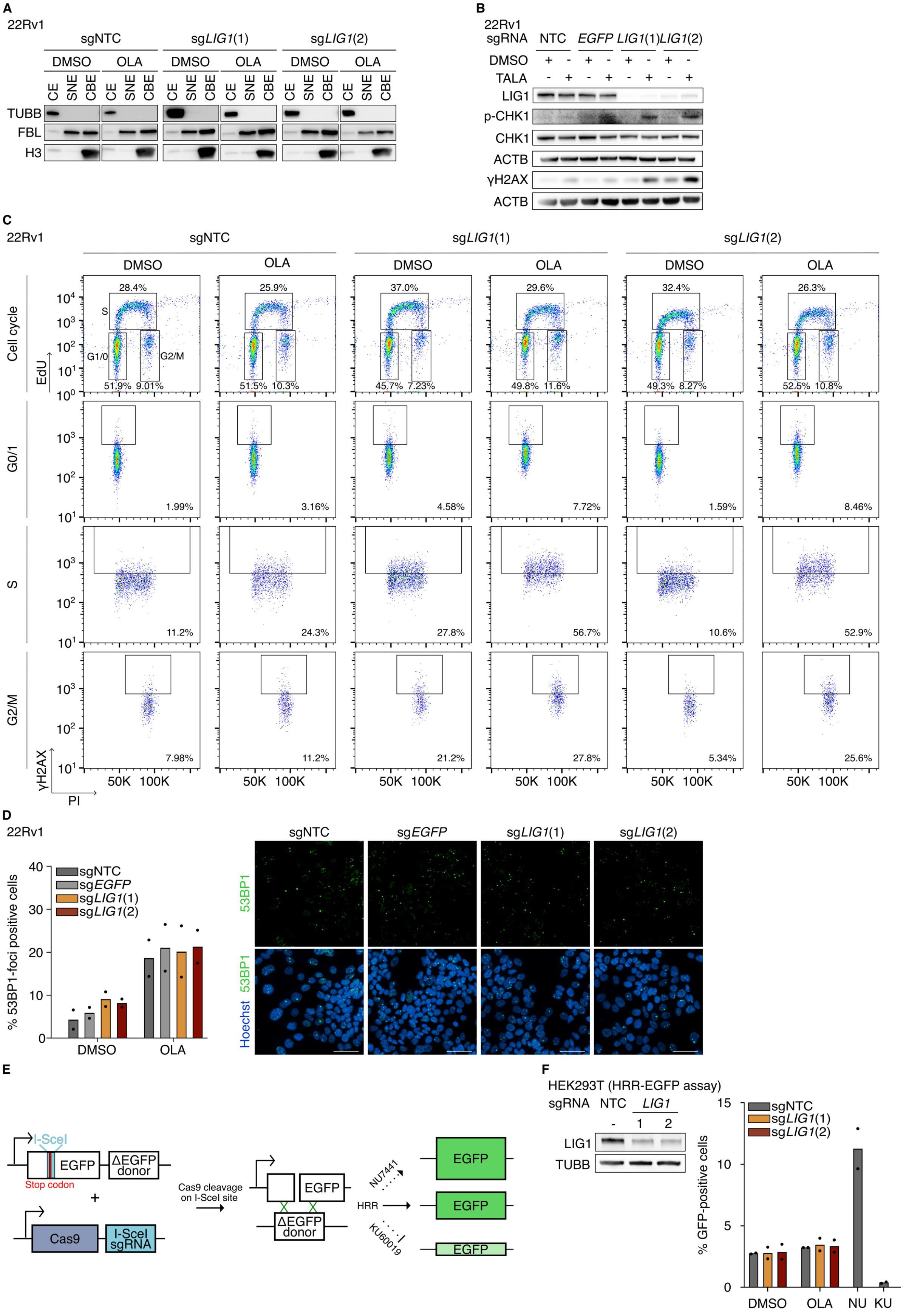
A
22Rv1
sgNTC
sgLIG1(1)
sgLIG1(2)
DMSO
OLA
CE
SNE
CBE
TUBB
FBL
H3
B
22Rv1
sgRNA
NTC
EGFP
LIG1(1)
LIG1(2)
DMSO
TALA
LIG1
p-CHK1
CHK1
ACTB
γH2AX
ACTB
C
22Rv1
sgNTC
sgLIG1(1)
sgLIG1(2)
DMSO
OLA
Cell cycle
G0/1
S
G2/M
EdU
γH2AX
PI
S
G1/0
G2/M
28.4%
51.9% 9.01%
25.9%
51.5% 10.3%
37.0%
45.7% 7.23%
29.6%
49.8% 11.6%
32.4%
49.3% 8.27%
26.3%
52.5% 10.8%
1.99%
3.16%
4.58%
7.72%
1.59%
8.46%
11.2%
24.3%
27.8%
56.7%
10.6%
52.9%
7.98%
11.2%
21.2%
27.8%
5.34%
25.6%
50K 100K
D
22Rv1
% 53BP1-foci positive cells
sgNTC
sgEGFP
sgLIG1(1)
sgLIG1(2)
DMSO
OLA
53BP1
Hoechst 53BP1
E
I-SceI
EGFP
ΔEGFP donor
Stop codon
Cas9
I-SceI sgRNA
Cas9 cleavage on I-SceI site
NU7441
HRR
KU60019
F
HEK293T (HRR-EGFP assay)
sgRNA NTC LIG1
- 1 2
LIG1
TUBB
% GFP-positive cells
sgNTC
sgLIG1(1)
sgLIG1(2)
DMSO
OLA
NU
KU

Supplemental Figure 4| *LIG1* loss and PARP inhibition induce DNA damage and do not impair NHEJ and HRR pathways.

A Immunoblot of TUBB, FBL and H3 in the cytoplasmic (CE), soluble nuclear (SNE), and chromatin-bound (CBE) extracts of 22Rv1 transduced with the indicated sgRNAs and treated for 3 days with DMSO, as control, or OLA. **B** Immunoblot of LIG1, p-CHK1, CHK1, γH2AX, and ACTB (used as loading control) in 22Rv1 transduced with the indicated sgRNAs and treated for 3 days with DMSO, as control, or TALA. **C** Representative images of EdU/PI and γH2AX/PI flow cytometry analysis. Numbers indicate the percentage of cells in the corresponding gate. 22Rv1 cells transduced with the indicated sgRNAs were treated as in A. **D** Quantification of cells with 10 or more foci and representative images of 53BP1 immunofluorescence. 22Rv1 transduced with the indicated sgRNAs were treated as in A. Data are presented as mean (n=2 biological replicates). Scale bar, 50 μm. **E** Schematic of HRR-EGFP assay. **F** Immunoblot of LIG1 and TUBB (used as loading control) in HEK293T-EGFP reporter cells transduced with the indicated sgRNAs and quantification of EGFP-positive HEK293T cells measured by flow cytometry after HRR-EGFP assays. HEK293T cells transduced with the indicated sgRNAs and the lentiviral vectors described in E were treated for 5 days with DMSO, as control, or OLA. HEK293T-sgNTC cells were treated for 5 days with the DNA-PK inhibitor NU7441 (NU, 1 μM) or the ATM inhibitor KU60019 (KU, 5μM) as controls for HRR activity. Data are presented as mean (n=2 biological replicates).

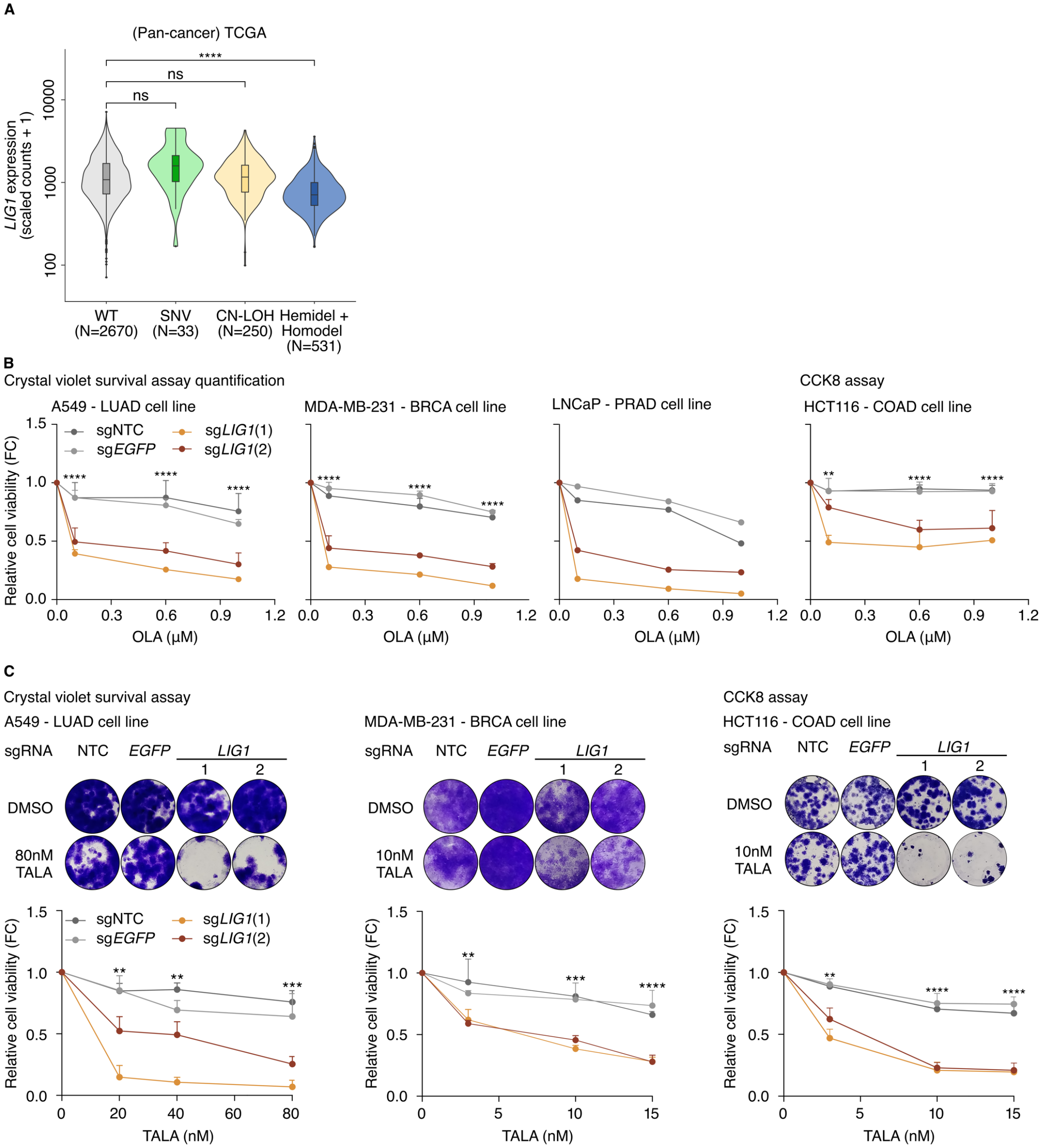

Supplemental Figure 5| *LIG1* loss sensitizes multiple tumor models to OLA and TALA treatment.

A Violin plot of *LIG1* expression in pan-cancer TCGA samples stratified by type of aberrations (n=3484 (7)). P-values were determined using a one-sided Wilcoxon signed-rank test. **B** Quantification of cell viability assay included in figure 5B. Cells transduced with the indicated sgRNAs were treated with DMSO, as control, or OLA at different concentrations for 8-12 days (n=2 biological replicates for LNCaP, n=3 technical replicates for the other cell lines). **C** Representative images of crystal violet assays and corresponding quantifications (HCT116 plot represents CCK8 assay results). Cells were transduced with the indicated sgRNAs and treated with DMSO, as control, or TALA at different concentrations for 12-14 days. Data are presented as mean ± SD (n=3 technical replicates). In B and C, p-values were determined using the two-way ANOVA and Bonferroni's multiple comparisons test on control (sgNTC and sgEGFP) and sg*LIG1* samples. ** p-value ≤ 0.01; *** p-value ≤ 0.001; **** p-value ≤ 0.0001. WT: wild type.

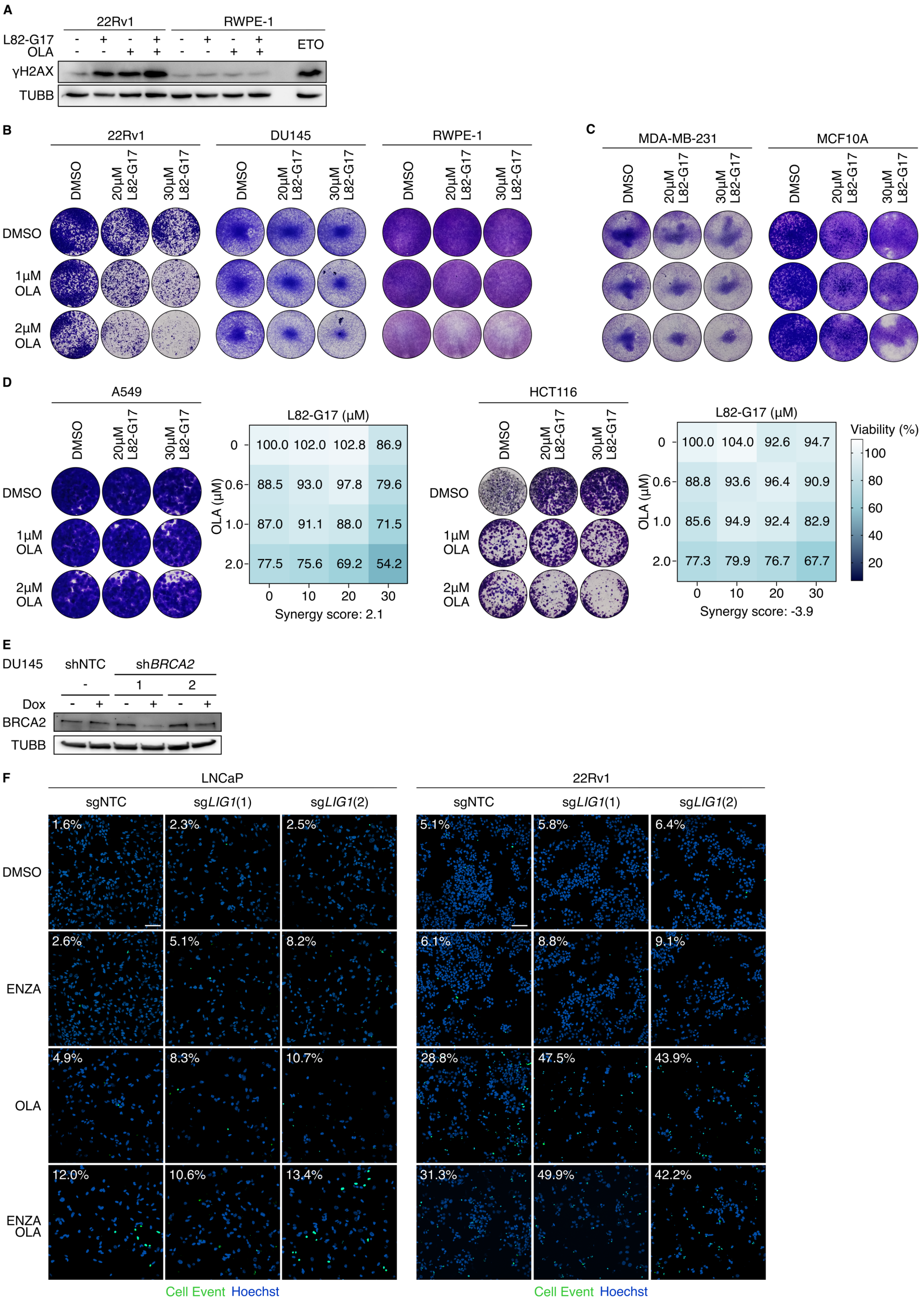
A
22Rv1
RWPE-1
L82-G17
OLA
ETO
γH2AX
TUBB
B
22Rv1
DU145
RWPE-1
DMSO
20µM L82-G17
30µM L82-G17
DMSO
1µM OLA
2µM OLA
C
MDA-MB-231
MCF10A
D
A549
HCT116
L82-G17 (µM)
OLA (µM)
0 100.0 102.0 102.8 86.9
0.6 88.5 93.0 97.8 79.6
1.0 87.0 91.1 88.0 71.5
2.0 77.5 75.6 69.2 54.2
0 10 20 30
Synergy score: 2.1
0 100.0 104.0 92.6 94.7
0.6 88.8 93.6 96.4 90.9
1.0 85.6 94.9 92.4 82.9
2.0 77.3 79.9 76.7 67.7
0 10 20 30
Synergy score: -3.9
Viability (%)
100
80
60
40
20
E
DU145
shNTC
shBRCA2
1
2
Dox
BRCA2
TUBB
F
LNCaP
22Rv1
sgNTC
sgLIG1(1)
sgLIG1(2)
DMSO
ENZA
OLA
ENZA OLA
1.6% 2.3% 2.5% 5.1% 5.8% 6.4%
2.6% 5.1% 8.2% 6.1% 8.8% 9.1%
4.9% 8.3% 10.7% 28.8% 47.5% 43.9%
12.0% 10.6% 13.4% 31.3% 49.9% 42.2%
Cell Event Hoechst
Cell Event Hoechst

Supplemental Figure 6| Combined pharmacological inhibition of LIG1 and PARP specifically reduces the viability of prostate and breast cancer cells.

A Immunoblot analysis of γH2AX and TUBB (used as loading control) in 22Rv1 and RWPE-1 cells treated with 2μM OLA and 40μM L82-G17 for 3 days (DMSO was used as control) and in RWPE-1 cells treated with 50μM ETO for 1 day. **B, C** Representative images of crystal violet assays included in figures 6B and C. **D** Representative images of cell viability assays and quantifications (crystal violet for A549 and CCK8 for HCT116). A549 and HCT116 were treated with OLA and L82-G17 for 12 and 8 days, respectively. Data are presented as mean (n=3 biological replicates). Synergy scores were calculated by using the HSA model. **E** Immunoblot analysis of BRCA2 and TUBB (used as loading control) in DU145 transduced with the indicated shRNAs and treated with 1μg/ml Dox or EtOH, as control, for 1 day. **F** Representative images and quantification (%) of apoptotic cells and bodies measured by CellEvent Caspase 3/7 assay in LNCaP and 22Rv1 cells transduced with the indicated sgRNA and treated with 1μM ENZA and 10μM OLA for 3 days. Scale bar, 100 μm. ETO: etoposide, Dox: doxycycline.

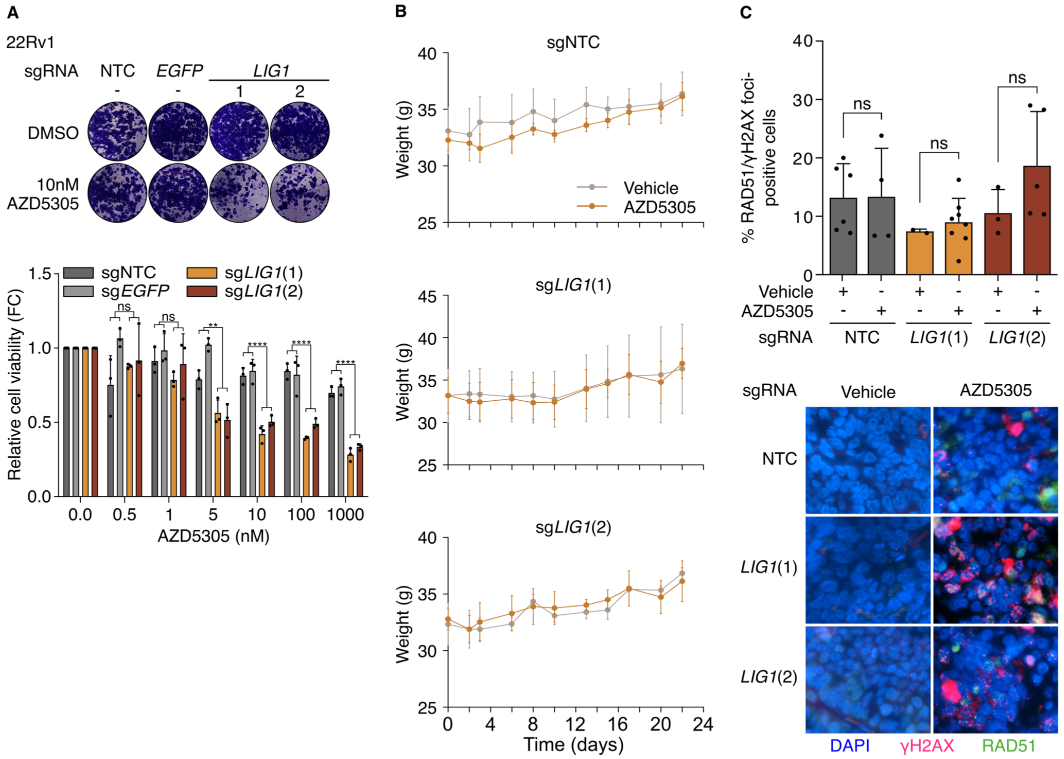

Supplemental Figure 7| *LIG1* loss sensitizes PCa cells to AZD5305.

A Representative images of crystal violet assays with corresponding quantification. 22Rv1 cells transduced with the indicated sgRNAs were treated with DMSO, as control, or AZD5305 at different concentrations for 14 days. Data are presented as mean ± SD (n=3 technical replicates). P-values were determined using the two-way ANOVA and Bonferroni's multiple comparisons test on control (sgNTC and sgEGFP) and sg*LIG1* samples. **B** Scatter plot of the mice body weight during treatment with vehicle or AZD5305 (0.25mg/kg). Data are presented as mean ± SD. **C** Percentage of cells with 5 or more γH2AX and RAD51 foci measured by immunofluorescence in FFPE xenograft tumor samples collected after the *in vivo* experiments described in figure 7A. Images are representative of FFPE xenografts tumor sections stained for γH2AX (red), RAD51 (green) and nucleus (DAPI, blue). Data are presented as mean ± SD. P-values were determined using a two-tailed unpaired t-test. ** p-value ≤ 0.01; *** p-value ≤ 0.001; **** p-value ≤ 0.0001.

References

1. Orlando F, et al. Allele-informed copy number evaluation of plasma DNA samples from metastatic prostate cancer patients: the PCF_SELECT consortium assay. *NAR Cancer*. 2022;4(2):zcac016.

2. Hart T, et al. High-Resolution CRISPR Screens Reveal Fitness Genes and Genotype-Specific Cancer Liabilities. *Cell*. 2015;163(6):1515–1526.

3. Meyers RM, et al. Computational correction of copy number effect improves specificity of CRISPR–Cas9 essentiality screens in cancer cells. *Nat Genet*. 2017;49(12):1779–1784.

4. Dempster JM, et al. Chronos: a cell population dynamics model of CRISPR experiments that improves inference of gene fitness effects. *Genome Biol*. 2021;22(1):343.

5. Wilks C, et al. recount3: summaries and queries for large-scale RNA-seq expression and splicing. *Genome Biol*. 2021;22(1):323.

6. Liu J, et al. An Integrated TCGA Pan-Cancer Clinical Data Resource to Drive High-Quality Survival Outcome Analytics. *Cell*. 2018;173(2):400-416.e11.

7. Ciani Y, et al. Allele-specific genomic data elucidate the role of somatic gain and copy-number neutral loss of heterozygosity in cancer. *Cell Syst*. 2022;13(2):183-193.e7.
