# Supplementary material for: CRISPR/Cas9 screens identify LIG1 as a sensitizer of PARP inhibitors in castration-resistant prostate cancer
Source: J Clin Invest. 2024 Dec 24;135(4):e179393. doi: 10.1172/JCI179393 (PMC11827843; doi:10.1172/JCI179393)

Full unedited blots for Figure 2A (left and middle panels)

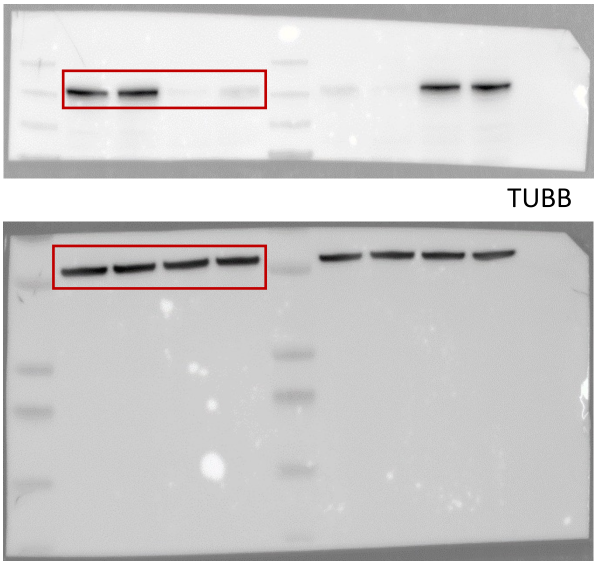

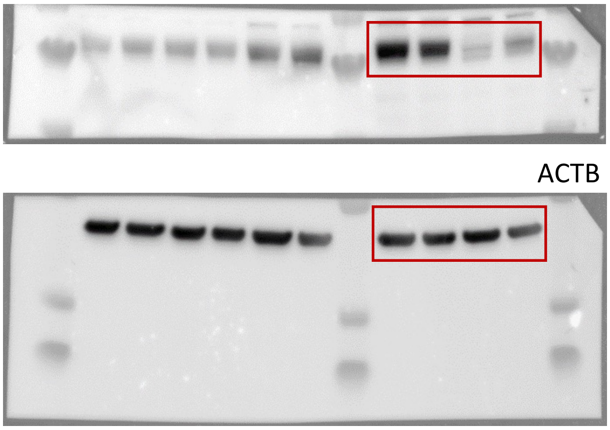

Full unedited blots for Figure 3A

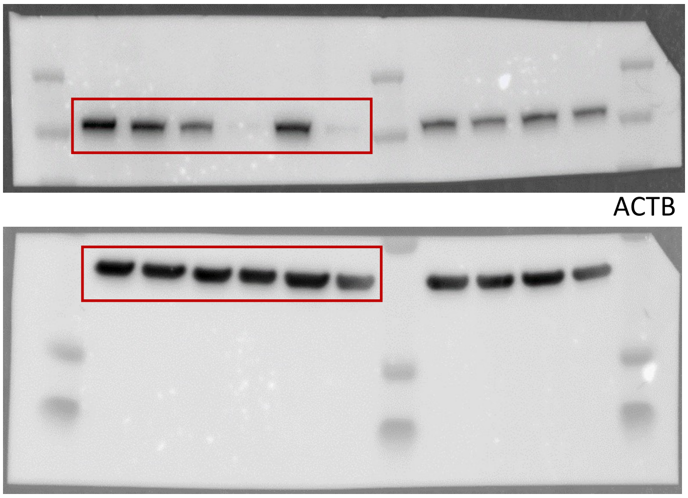

Full unedited blots for Figure 3C

KDa

PARP and cleaved PARP

190
115
80
70

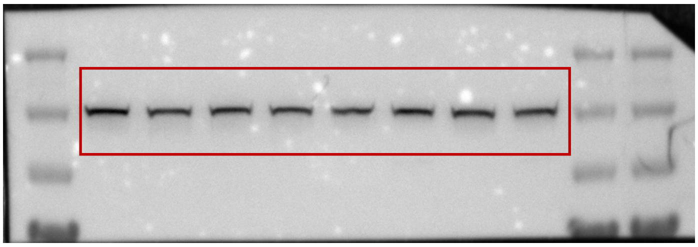

cleaved PARP

80
70

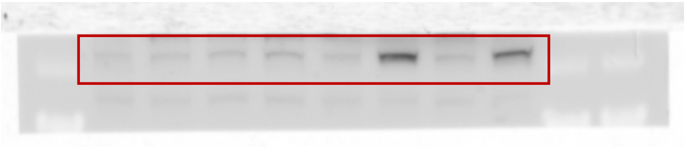

TUBB

70
50
30

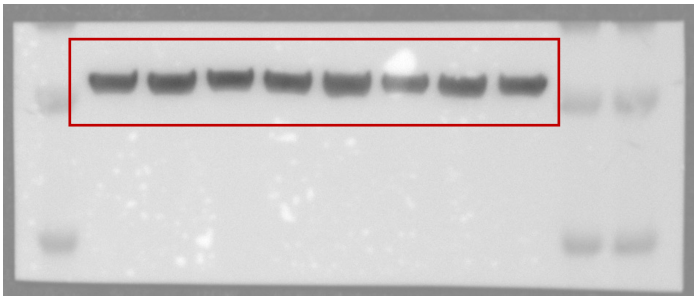

ACTB

70
50
30

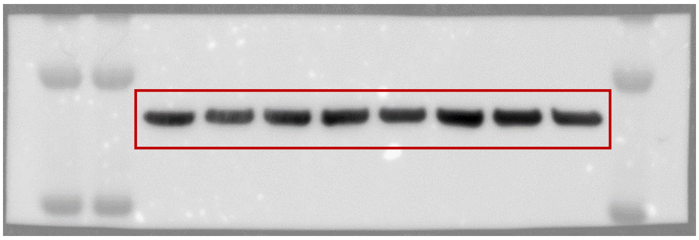

Cleaved Caspase-3

25
15
10

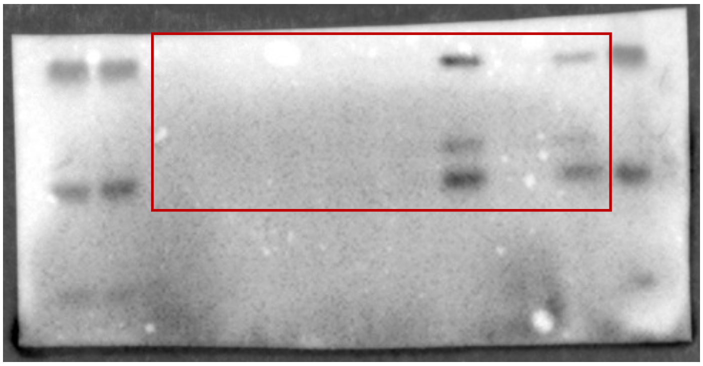

Full unedited blots for Supplemental Figure 3C

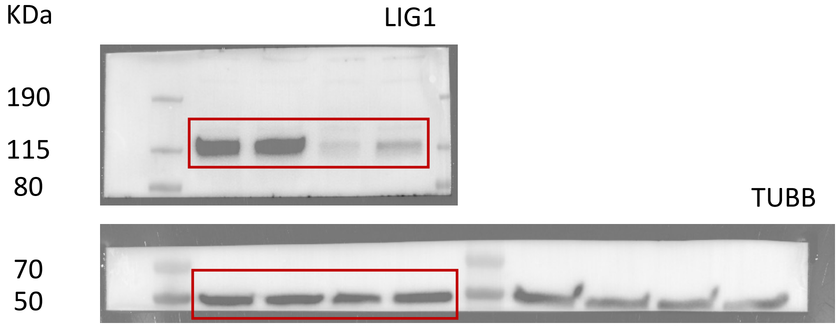

Full unedited blots for Supplemental Figure 3F

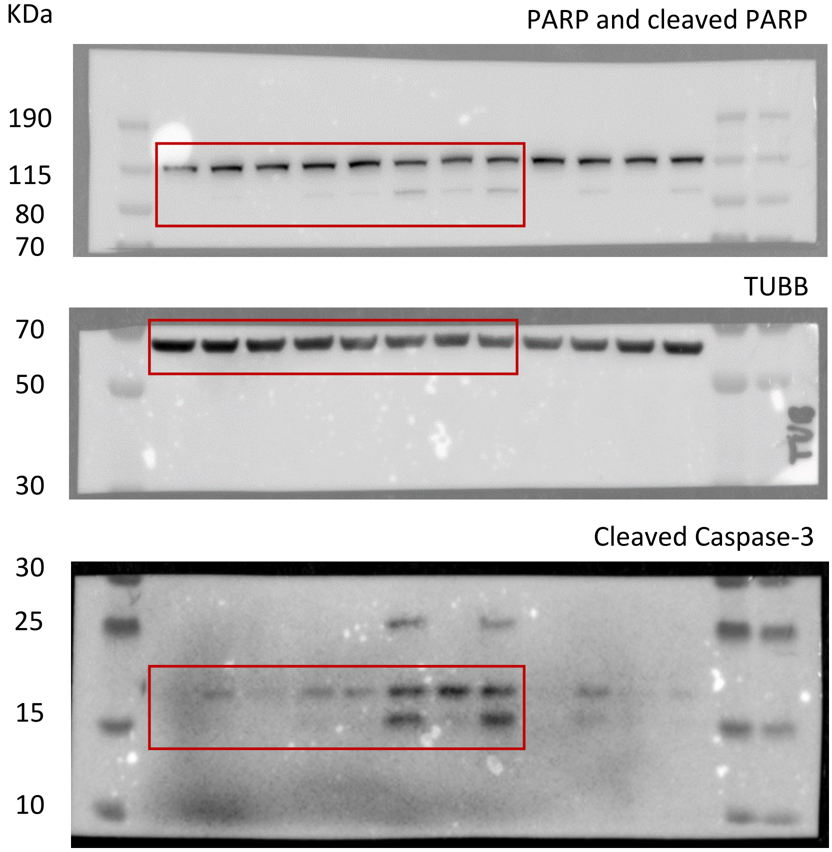

Full unedited blots for Figure 4A

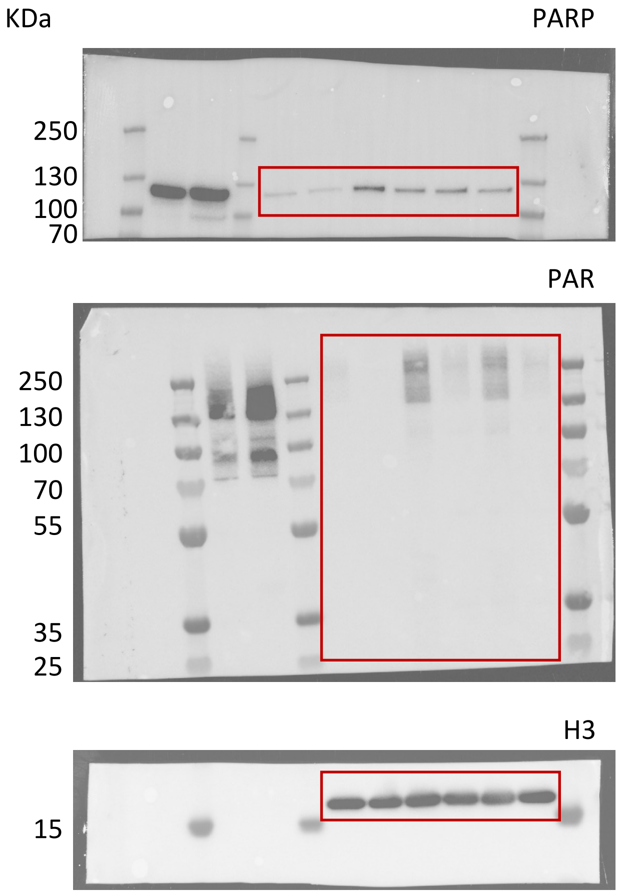

Full unedited blots for Figure 4D

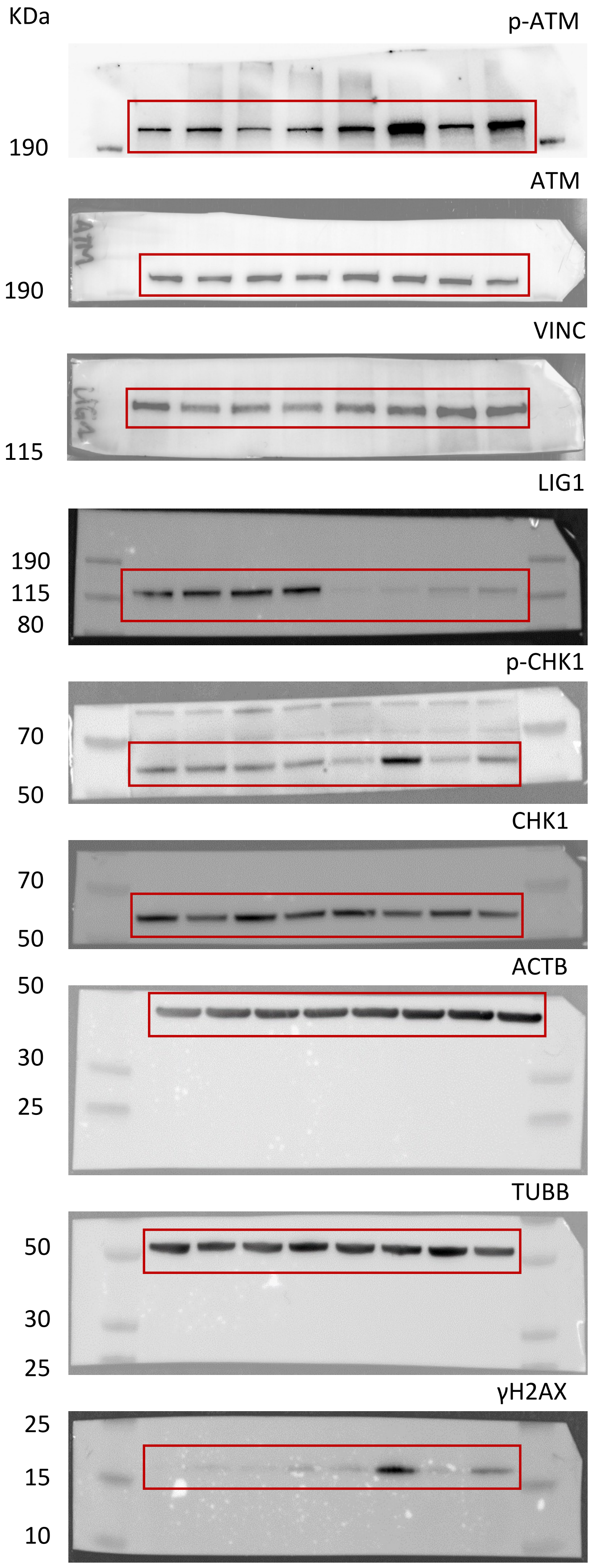

Full unedited blots for Supplemental Figure 4A

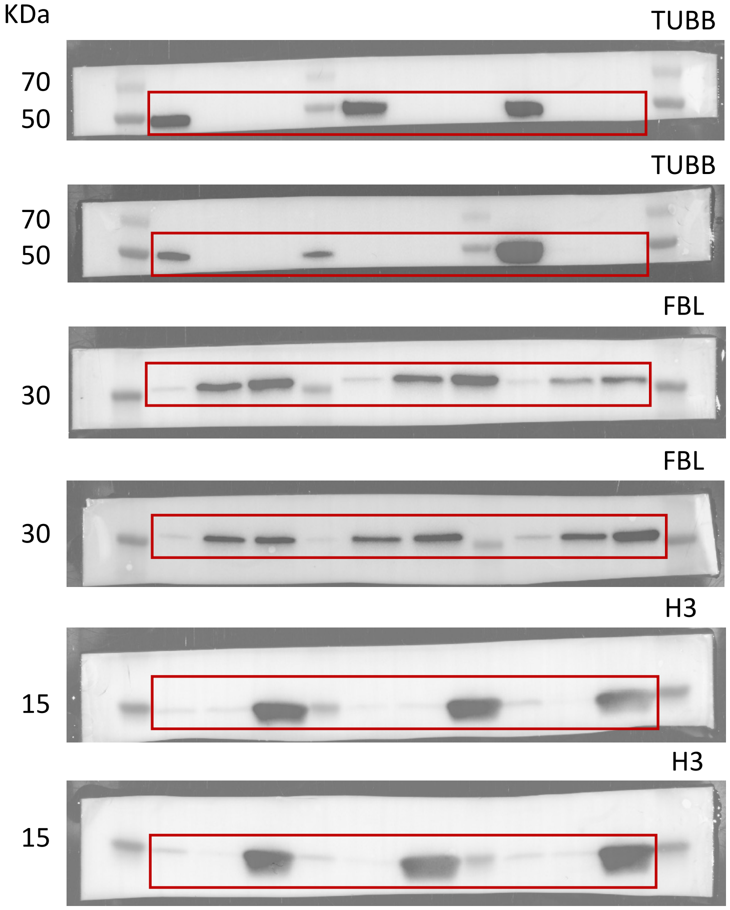

Full unedited blots for Supplemental Figure 4B

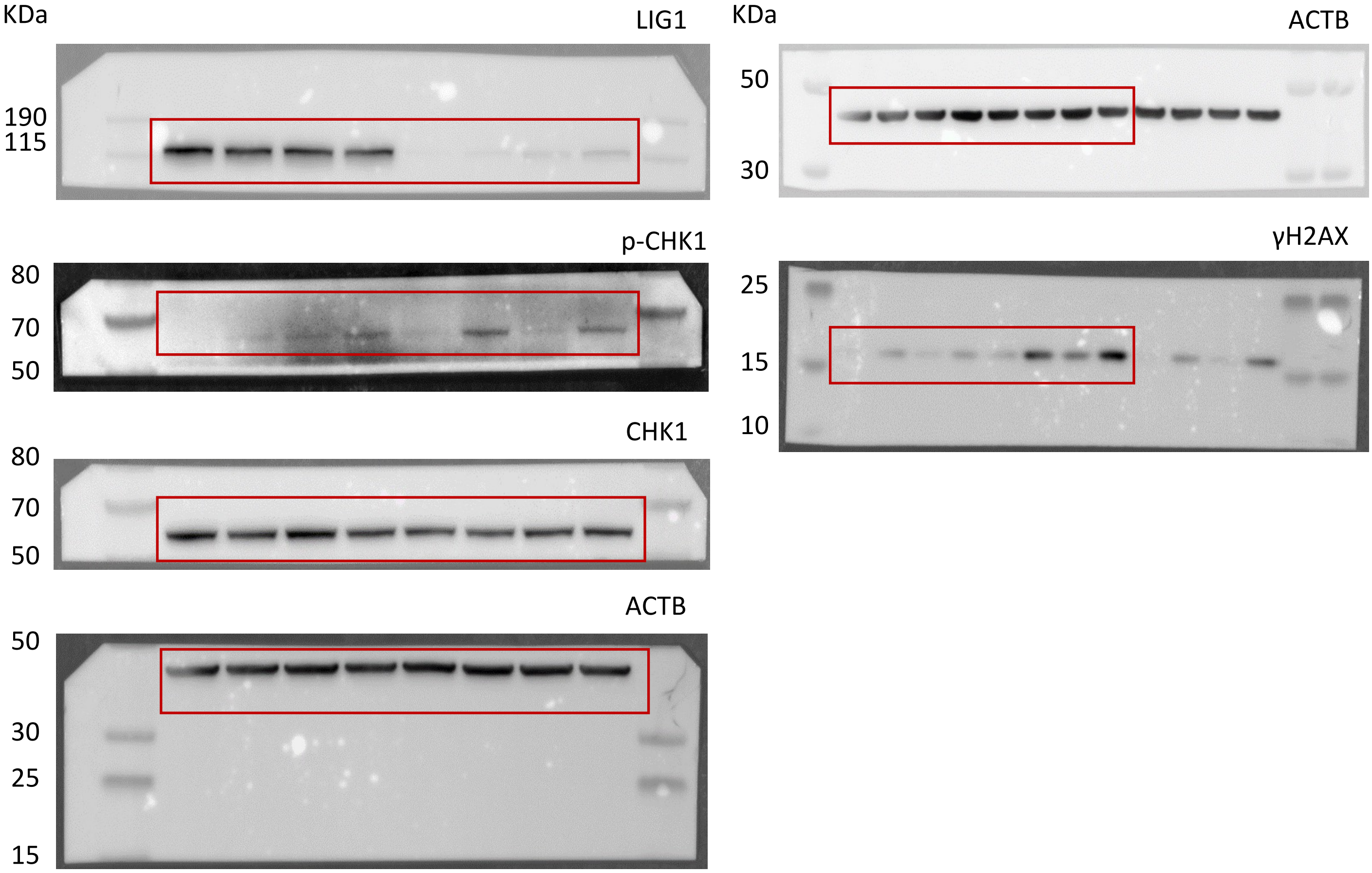

Full unedited blots for Supplemental Figure 4F

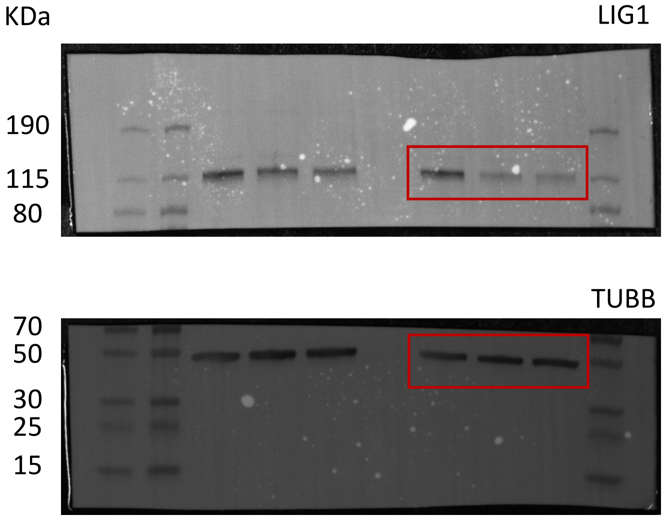

Full unedited blots for Supplemental Figure 6A

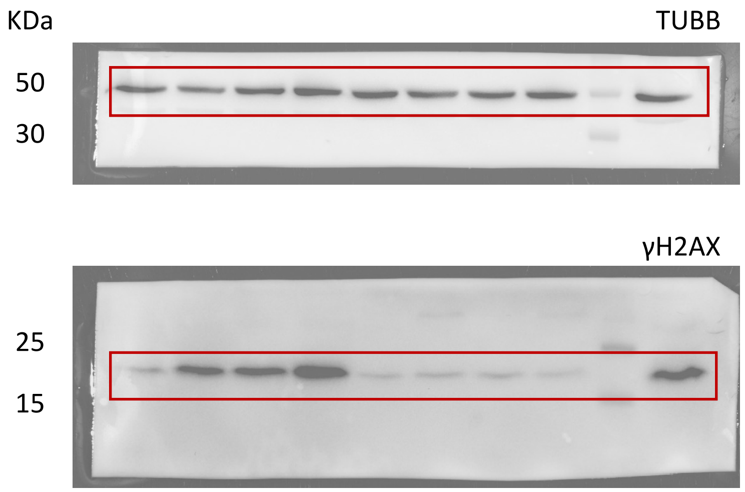

Full unedited blots for Supplemental Figure 6E

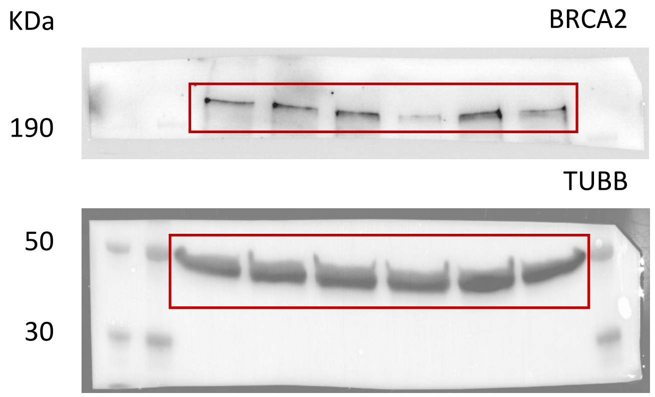

Full unedited blots for Figure 7B

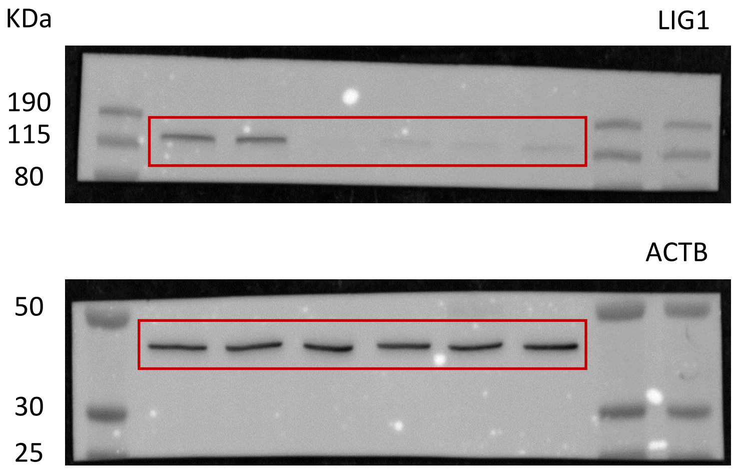

Supplement: Unedited blot and gel images [file jci-135-179393-s163.pdf]
